# Arylsulfatase K attenuates airway epithelial cell senescence in COPD by regulating parkin-mediated mitophagy

**DOI:** 10.1016/j.redox.2025.103793

**Published:** 2025-07-31

**Authors:** Ruonan Yang, Yuan Zhan, Zhesong Deng, Jiaheng Zhang, Shanshan Chen, Yating Zhang, Hao Fu, Xiangling Meng, Jixing Wu, Yiya Gu, Qian Huang, Congyi Wang, Jungang Xie

**Affiliations:** a Department of Respiratory and Critical Care Medicine, National Clinical Research Center of Respiratory Disease, Key Laboratory of Pulmonary Diseases of Health Ministry, Tongji Hospital, Tongji Medical College and State Key Laboratory for Diagnosis and Treatment of Severe Zoonotic Infectious Disease, Huazhong University of Science and Technology, Wuhan, Hubei, 430030, China; b Department of Respiratory and Critical Care Medicine, The First Affiliated Hospital of Chongqing Medical University, Chongqing, China; c Department of Respiratory and Critical Care Medicine, Fuzhou University Affiliated Provincial Hospital, Fuzhou, China

**Keywords:** Chronic obstructive pulmonary disease, Cigarette smoke, Airway epithelial cell senescence, Mitophagy, Arylsulfatase K

## Abstract

Chronic obstructive pulmonary disease (COPD) is a heterogeneous lung condition characterized by irreversible airflow limitation, primarily due to cigarette smoke (CS) exposure. Emerging research underscores the pivotal role of cellular senescence in the pathogenesis of COPD. The arylsulfatase family, known for its involvement in various age-related diseases, has yet to be investigated in the context of COPD. This study investigated the role of the arylsulfatase family, particularly ARSK, in COPD pathogenesis. Bioinformatics analysis and clinical validation revealed significantly reduced ARSK expression in COPD patients' lungs, especially in airway epithelium. ARSK overexpression alleviated CS-induced epithelial cellular senescence and improved mitophagy and mitochondrial function, while ARSK knockdown had an opposite effect. In *vivo*, Arsk-AAV administration relieved lung senescence and impaired lung function upon CS exposure, whereas airway-specific Arsk knockout aggravated these effects. Mechanistically, ARSK interacted with Parkin (PRKN) to regulate the phosphorylation of PRKN at serine 65 and subsequent mitophagy, thus attenuating cellular senescence. Additionally, the androgen receptor (AR) was identified as a transcription factor binding to the ARSK promoter, modulating its expression. These findings highlight the protective role of ARSK against epithelial cellular senescence, offering a potential therapeutic target for COPD.

## Graphical abstract

Cigarette smoke impairs the expression of AR in airway epithelial cells, thereby diminshing the transcription and expression of ARSK. This reduction disrupts the interaction between ARSK and PRKN, facilitating the phosphorylation of PRKN at serine 65 and its subsequent translocation to mitochondria. This cascade of events triggers excessive mitophagy, leading to mitochondrial dysfunction and subsequent cellular senescence, which drives the progression of COPD.

**Figure:**
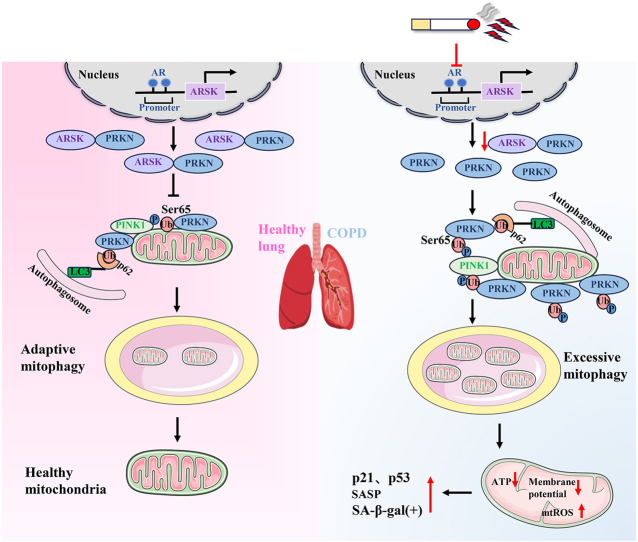

## Abbreviations

ARSK: arylsulfatase K
BALF: bronchoalveolar lavage fluid
COPD: chronic obstructive pulmonary disease
CS: cigarette smoke
CSE: cigarette smoke extract
DEGs: differentially expressed genes
ELISA: Enzyme-linked immunosorbent assay
FEV1: forced expiratory volume in 1 s
FEV0.1s: forced expiratory volume in 0.1 s
FVC: forced vital capacity
HBE: human bronchial epithelial cell
IL-: interleukin-
IP: immunoprecipitation
LC3B: Microtubule-associated protein 1 light chain 3β
LIE: liensinine
MLI: mean linear intercept
MMP: mitochondrial membrane potential
PINK1: PTEN induced putative kinase 1
PRKN/Parkin: parkin RBR E3 ubiquitin protein ligase
SA-β-gal: senescence-associated-b-galactosidase
SASP: senescence-associated secretory phenotype
Ser65: serine 65
TOMM20: translocase of outer mitochondrial membrane 20.

## 1 Introduction

Chronic obstructive pulmonary disease (COPD), a respiratory disorder marked by long-term breathing difficulties and progressive airflow obstruction, currently ranks as the third most prevalent cause of mortality worldwide and continues to exert significant socioeconomic pressures across global healthcare systems [1,2]. Among numerous risk factors, cigarette smoke (CS) stands out as a predominant risk factor for COPD [3,4]. Upon encountering CS, airway epithelial cells, acting as the frontline guardians of respiratory system, swiftly react to injury and trigger a cascade of complex inflammatory responses, which fosters the progression of COPD [5]. Thus, the functional homeostasis of airway epithelial cells is of great importance for maintaining a healthy lung environment.

Cellular senescence, particularly of airway epithelial cells, has been identified as a significant driving force in the pathogenesis of COPD, which is considered an age-related disease [6,7]. Senescent cells are recognized for releasing various proinflammatory cytokines, chemokines and matrix metalloproteinases, collectively termed as senescence associated secretory phenotype (SASP) [8]. SASP can induce further senescence in both the cells themselves and surrounding cells, thus aggravating and propagating cellular senescence [9]. Targeting cellular senescence with therapeutic interventions appears to be a promising approach to mitigate the progression of COPD [10,11].

Mitophagy, is fundamental to preserving mitochondria and intracellular homeostasis by selectively wrapping and degrading damaged mitochondria [12]. The PTEN induced kinase 1/parkin RBR E3 ubiquitin protein ligase (PINK1/Parkin)-mediated mitophagy is the most common type to initiate the elimination of dysfunctional mitochondria [13]. To initiate mitophagy, PINK1 phosphorylated Parkin and ubiquitin at residue Serine 65 (Ser65). Parkin then constructs ubiquitin chains on mitochondrial outer membrane proteins, which serve to recruit autophagy receptors [14,15]. Abnormal mitophagy has been implicated in oxidative stress, inflammation and cellular senescence, which are central to the pathogenesis of COPD [16,17].

Arylsulfatase is a family of lysosomal enzyme which is involved in regulations of hormone, cell signaling pathway, and cellular degradation [18,19]. Deficiencies or mutations in arylsulfatase family members have been linked to the presence of age-related diseases, such as Alzheimer's disease and Parkinson's disease [20,21]. Moreover, altered expression of arylsulfatase family member A (ARSA) has been observed in senescent fibroblasts and contributed to SASP [22]. However, the expression patterns and functions of arylsulfatase family members in COPD remain unexplored.

In this study, we are committed to investigating which and how the arylsulfatase family members differentially express and regulate cell senescence in COPD. Our findings indicated that decreased ARSK expression may be pivotal to COPD pathogenesis, via increasing the phosphorylation of Parkin at Serine65 to exacerbate excessive mitophagy and subsequent senescence in airway epithelial cells. The crucial findings suggest that ARSK may serve as a promising therapeutic target for mitigating the pathogenic damage of COPD.

## 2 Materials and methods

### 2.1 Bioinformatic analysis

The dataset GSE38974, sourced from the Gene Expression Omnibus (GEO, https://www.ncbi.nlm.nih.gov./gds), comprised 9 healthy controls and 23 COPD patients [23]. Genes with a log fold change exceeding 0.94 and a P-value below 0.05 were identified as differentially expressed genes (DEGs). Subsequent bioinformatic analysis was conducted utilizing R studio, version 4.1.2.

### 2.2 Subjects

Lung tissue samples were collected from three distinct groups: 18 non-smokers, 18 healthy smokers, and 20 COPD patients, all sourced from Tongji Hospital, Wuhan, China (Clinical characteristics of subjects were showed in Table S1). COPD diagnosis was made in accordance with the guidelines set by the Global Initiative of Chronic Obstructive Pulmonary Disease (GOLD). Participants were categorized into three groups based on their pulmonary function and smoking background. Individuals with other chronic respiratory conditions like asthma or interstitial lung disease, those who had undergone steroid therapy recently, and those with a history of other types of cancers were not included in the study. The research protocol was sanctioned by the ethics committee of Tongji Hospital (TJ-IRB20210346) in Wuhan, China. Prior to participation, each subject provided written consent.

### 2.3 Mouse model

To generate mice with airway bronchial cell-specific knockdown of Arsk (Scgb1a1-Cre^+^- Arsk^flox/flox^ mice, hereinafter referred to as Cre^+^ mice), we acquired Arsk^flox/flox^ mice and Scgb1a1-Cre^+^ mice from the Gempharmatech (Nanjing, China). We then bred the Arsk^flox/flox^ mice with Scgb1a1-Cre^+^ mice to produce offspring. The littermates Scgb1a1-Cre^-^- Arsk^flox/flox^ mice (hereinafter referred to as Cre^−^ mice) were used as controls. For the overexpression mice, we purchased wildtype male C57BL/6 mice from the same source. Mice were intratracheally administered with Arsk/Con-AAV2/9 virus (total amount of titer 2 × 10^11^ μg per mouse) 21 days prior to CS exposure.

All mice (8–12 weeks old, male) were housed in microisolator cages under a 12-h light/dark cycle, with controlled temperature (22 ± 1 °C) and humidity (55 ± 5 %) within the specific pathogen-free (SPF) animal facility at the Tongji hospital. They were subjected to either ambient air or CS for roughly 3 h each day (12 cigarettes over 45 min per session, with 4 sessions daily) and 5 days per week over a 3-month period. The exposure chamber had a volume of 45 L, and the total particulate matter concentration was maintained between 80 and 120 mg/m^3^. Marlboro Red cigarettes were used for all exposure experiments. Dynamic ventilation was employed throughout the exposure period to ensure consistent exposure conditions and to safeguard animal welfare. In the liensinine (LIE) intervention experiment, Cre^+^ mice were orally administrated with LIE (5 mg/kg/d, MCE) or an equivalent volume of PBS throughout the remaining 4 weeks of air/CS exposure. All animal experimental procedures were authorized by the ethics committee of the Tongji Hospital (TJH-202207006).

### 2.4 Pulmonary function test

Briefly, the pulmonary function of all mice was assessed using the FlexiVent system (SCIREQ, Canada) while they were under general anesthesia, which was induced via intraperitoneal injection of 1 % pentobarbital sodium at a dose of 10 ml/kg body weight. Pulmonary function parameters FEV0.1s/FVC was calculated.

### 2.5 Reagents and antibodies

The preparation method of cigarette smoke extract (CSE) was under the previous description [24]. Liensinine (LIE), BafilomycinA1(BafA1), BMS-564924 were purchased from MedChem Express (Monmouth Junction, NJ, USA). Antibodies against β-Actin, GAPDH, p21, p53, PINK1, Parkin, TOMM20, p62, LC3B, LC3C, LAMP1, LAMP2, TFEB, Galectin3, CTSB, Flag and His were purchased from Proteintech. Antibodies against *p*-Ser65-Parkin was purchased from Affinity. Antibodies against IgG was purchased from Cell Signaling Technology (Danvers, MA, USA). Goat Anti-Mouse IgG LCS, Acridine orange hydrochloride were purchased from Promoter (Wuhan, China).

### 2.6 Cell culture and treatment

Human bronchial epithelial cells HBE4-E6/E7 (ATCC, CRL-2078) were cultured in 1640 medium supplemented with 10 % fetal bovine serum. The cells were maintained at 37 °C in a humidified incubator with 5 % CO2 and exposed to varying concentrations of CSE for different durations. For overexpression experiment, cells were transfected with ARSK plasmid (5′-CTAGCTAGCCACCATGCTACTGCTGTGGGTGTCG GTGG-3′) or Parkin S65E plasmid (5′- GACCTGGATCAGCAGGCCATTGTTCAC ATTGT- 3′). For knockdown experiment, cells were transfected with small interfering RNA (siRNA) targeting ARSK (5′-GGACTATACTTCAGGACAT-3′) (RiboBio, Guangzhou, China) and RELA (5′-CCGGATTGAGGAGAAACGTAA-3′) (Sangon Biotech, Shanghai, China) or negative control using lipofectamine 3000. In pharmacological experiments, HBE cells were treated with 40uM LIE or 20 nM BMS-564924 for 24h.

### 2.7 Histopathology and immunofluorescence analysis

Hematoxylin and eosin (HE) staining was applied to the fixed lung sections from mice. The degree of peribronchial inflammation and the mean linear intercept (MLI) were evaluated based on a minimum of five random photomicrographs per mouse. The calculations of peribronchial inflammation and MLI, suggesting the condition of inflammation and emphysema respectively, were conducted as previously reported [25].

For the detection of mitophagy *in vitro*, HBE cells on cells slides were incubated with TOMM20 (1:200, proteintech) and LC3B (1:200, proteintech) primary antibody and observed in a confocal microscope. In immunofluorescence experiment, mouse lung sections were incubated with anti-p21 or anti-p53 primary antibody (1:200, Proteintech), followed by the application of fluorescent secondary antibodies of corresponding species (Servicebio, Wuhan, China).

### 2.8 Fluorescence in situ hybridization

Fluorescence in situ hybridization (FISH) was conducted to assess ARSK expression in fixed human lung sections, mouse lung sections, and HBE cells on cell slides at Pinuofei Biological Technology Company (Wuhan, China). A digoxigenin-labeled locked nucleic acid (LNA) probe specific for ARSK was utilized, with the sequence UUGUCCACGCUUCCACACGAUUA. The probe/target complex was visualized following sequential rounds of horseradish peroxidase (HRP)-mediated tyramide signal amplification (TSA) reactions.

### 2.9 Real-time qPCR

Trizol reagent (Takara, Japan) was used for the extraction of total RNA from lung tissues and HBE cells. Following reverse transcription with the cDNA RT PCR Kit (Takara, Japan), quantitative RT-PCR was conducted using SYBR Premix Ex Taq (Takara, Japan). Relative expression levels of target genes were normalized to β-Actin.

The primer sequences used in this study were displayed in TableS2.

### 2.10 Western blotting

Total protein extraction from lung tissues and HBE cells was performed using RIPA lysis buffer supplemented with phosphatase inhibitors (Promotor, Wuhan, China). The mitochondrial protein was extracted using Mitochondrial Isolation and Protein Extraction Kit (PK10016, Proteintech) according to manufacturer's instructions. The proteins were separated by 10 % or 12 % SDS-PAGE, transferred onto polyvinylidene fluoride (PVDF) membranes and blocked for 1–2 h in 5 % milk. Following an overnight incubation with primary antibodies at 4 °C, the membranes were visualized using chemiluminescent procedure (Bio-Rad, CA, USA).

### 2.11 ELISA

ELISA kits for human IL6, IL-8 and mouse IL-6, KC were purchased from R&D systems. ELISA kits for human and mouse IL-1β were purchased from Dakewei Bio engineering (Shenzhen, China). After the collection of cells culture supernatant and bronchoalveolar lavage fluid (BALF) of mice, the levels of above factors were determined following the manufacturer's instructions.

### 2.12 SA-β-gal assay

After washing with PBS and fixation in β-galactosidase fixation solution for 15 min, we stained HBE cells or frozen sections of mouse lung with SA-β-gal staining kit (Solarbio, Beijing, China) at 37 °C overnight.

### 2.13 Autophagic flux detection

HBE cells were seeded into 24-well cell plates and were infected with mRFP-eGFP-LC3B adenovirus (Genechem, Shanghai, China). After 24 h, cells were washed with PBS and fixed with 4 % paraformaldehyde, following the staining with DAPI for 10 min. Autophagosome (yellow puncta) and autolysosome (red puncta) were visualized by a confocal microscope (Zeiss LSM900 Airyscan).

### 2.14 Lysosomal integrity assessment by acridine orange staining

Acridine orange (AO) staining assay was performed to test the integrity of lysosomes. HBE cells were seeded into 24-well plates. Following experimental treatments, cells were incubated with AO (5 μM) in complete medium at 37 °C for 15 min under protected light conditions. Fluorescence imaging was immediately performed by a fluorescence microscope ((Olympus, Melville, NY, USA).

### 2.15 Lysotracker staining and lysosomal acidity measurement

Lysotracker red (Beyotime) and LysoSensor Green DND-189 (Invitrogen) were added into the culture medium to a final concentration of 50 nM and 2 μM, respectively. Then cells were incubated at 37 °C for 15 min and washed three times with culture medium. The images were acquired using a confocal microscope (Zeiss LSM 900 Airyscan).

### 2.16 Transmission electron microscopy

Fresh bronchia tissue (1–3 mm^3^) and human bronchial epithelial (HBE) cells were fixed at room temperature for 30 min and then transferred to 4 °C for 24 h. The samples were subsequently postfixed in 1 % osmium tetroxide and dehydrated using a graded ethanol series. Ultrathin sections were double-stained with uranyl acetate and lead citrate. Images were captured using a transmission electron microscope (HT7800, Hitachi).

### 2.17 Intracellular ROS analysis

HBE cells were grown in 12-well plates and subjected to transfection with siRNA or plasmid following by the treatment of CSE or LIE for 12 h. Subsequently, the cells were incubated with the DCFH-DA (Beyotime, China) working solution at 37 °C for 30 min. The fluorescence intensity of the cells was detected by Flow cytometry (BD Biosciences).

### 2.18 Mitochondrial reactive oxygen species (mtROS) analysis

HBE cells were grown in 12-well plates and stained with MitoSOX Red (Invitrogen) following the manufacturers’ instructions. The intensity of MitoSOX Red was observed by a fluorescence microscope ((Olympus, Melville, NY, USA).

### 2.19 Mitochondrial transmembrane potential measurement

In accordance with the manufacturers’ instructions, we assessed the mitochondrial transmembrane potential (MMP) in HBE cells by staining with JC-1 fluorescence dye (Beyotime, China) after treatment. Healthy mitochondria with a high membrane potential emit red fluorescence, while those with a reduced membrane potential emit green fluorescence. The percentage of green fluorescence, as detected by flow cytometry (BD Biosciences), was then plotted on a statistical graph to represent the changes in MMP.

### 2.20 Coimmunoprecipitation assay

Magnetic beads were purchased from MedChem Express (Monmouth Junction, NJ, USA). HBE cells infected with ARSK/CON-AAV were plated into 10-cm cell culture dishes and exposed to CSE for 24h. The whole cell lysates were extracted with NP40 lysis buffer supplemented with phosphatase inhibitors. After centrifugation, the supernatants were pre-cleared with magnetic beads for 2h at 4 °C. Then, the supernatants were incubated with either anti-His or anti-IgG antibody to allow the formation of immune complexes overnight at 4 °C. The next day, the complexes were immunoprecipitated using magnetic beads for an additional 2 h at 4 °C. Finally, the immune complexes were eluted from the beads and analyzed by Western blot.

### 2.21 Detection of ATP

A firefly luciferase-based ATP assay kit (Beyotime, China) was used to measure ATP content in the HBE cells following the manufacturers’ instructions. HBE cells were cultured in 6-well plates. The supernatants were collected by centrifugation after the treatment of ATP detection lysis. Then, the supernatants were mixed with the ATP detection working solution in a black 96-well plate. The levels of ATP content were detected by a luminometer.

### 2.22 RNA sequence and data analysis

HBE cells were grown in 12-well plates and transfected with siRNA of ARSK or negative control. Total RNA was extracted and sent to perform RNA-Seq at Linjian technology company (Wuhan, China) after the identification of knockdown of ARSK. DEGs were determined based on the parameters of |logFC| >0.5 and P-value <0.05. Gene Ontology (GO) analysis and Kyoto Encyclopedia of Genes and Genomes (KEGG) analysis were performed using cluster Profiler version 3.8.

### 2.23 Chromatin immunoprecipitation assay

ChIP assay was conducted using the ChIP Assay Kit (CST, #6003) according to the manufacturers’ protocol. HBE cells were seeded into 15-cm cell culture dishes and transfected with AR plasmid or negative control followed by CSE treatment for 24h. HBE cells were then cross-linked with 1 % formaldehyde (Sigma-F8775). The chromatin was extracted and cleaved into fragmentations under the protocol provided. Then, 10ug prepared chromatin fragmentation solution was incubated with anti-Flag (1:50) or normal rabbit immunoglobulin G (IgG) overnight at 4 °C. The binding DNA was used for ChIP-PCR. The amplified products were resolved on a 3 % agarose gel to identify the specific DNA fragments associated with the immunoprecipitated protein. The primer sequence to detect amplified products was TTACAGGCGTGAGCCACCGCGCCCAACACTGTTTACGTTTTTGCATGCTCCCGCAGATCTTTATCATGCTGCTTGAGTAACAATTCTGCTACACTAACAGATAAACTAACTGCAGACAGAGCATTTAGTGAGTTACGGCACAGACTCTTCAATTTTCAGTCGTGCTCTGACTGGCTCCCTGTGTAAACTCAGGCCAGTCATTTAACCTCTCTAAGCCTGTTTTCCTATCTGCAATATGGT.

### 2.24 Dual luciferase gene reporter assay

HBE cells were grown in 24-well plate. The full-length ARSK promoter, in either wild-type or mutant form, was cloned into pGL3-basic vectors (Genecreate, Wuhan, China) and co-transfected with or without AR overexpression vector later. Forty-eight hours post-transfection, the relative activity was quantified by the Dual Luciferase Reporter Assay Kit (Vazyme, China).

### 2.25 Statistical analysis

GraphPad Prism 8.0.2 Software (GraphPad Software Inc., San Diego, CA) was used for Statistical analysis. Data were expressed as mean ± SD. The differences between groups were analyzed using Student's *t*-test or one-way ANOVA. P-value less than 0.05 was defined as statistical significance.

## 3 Results

### 3.1 Decreased expression of ARSK in COPD patients, COPD model mice and CSE-treated bronchial epithelial cells

**Fig. 1:**
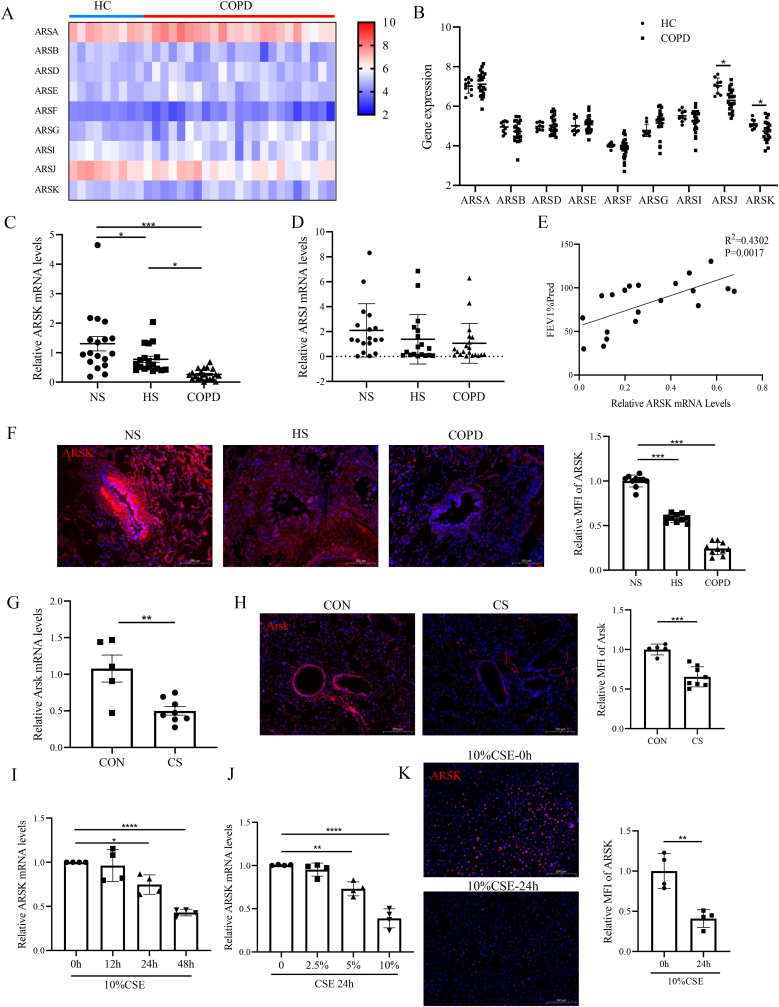
ARSK was downregulated in COPD patients, CS-exposed mice and CSE-treated HBE cells. (**A and B**) The heatmap and Gene expression of ARSK family members from GSE38974 dataset with 23 COPD patients and 9 healthy controls. (**C and D**) The relative mRNA expression of ARSK and ARSJ in collected lung tissues from non-somkers (n = 18), healthy-smokers (n = 18) and COPD patients (n = 20). (**E**) Correlation analysis between ARSK mRNA levels and lung function of COPD patients. (**F**) Representative FISH images and quantitative data of relative mean fluorescence intensity(MFI) against ARSK mRNA in lung sections of non-smokers, healthy smokers and COPD patients. Scale bars = 200μm, magnification = 200x, Red color-ARSK, Blue color-DAPI. (**G**) The relative mRNA levels of ARSK in lung tissues of air-exposed mice(n = 5) and CS-exposed mice(n = 8). (**H**) Representative FISH images and quantitative data of MFI for Arsk in mouse lung sections. Scale bars = 200μm, magnification = 200x, Red color-Arsk, Blue color-DAPI. (**I and J**) The relative ARSK mRNA expression in CSE-induced HBE cells under different concentrations and durations.(**K**) Representative FISH images and quantitative data of MFI for ARSK in HBE cells. Scale bars = 200μm, magnification = 200x, Red color-ARSK, Blue color-DAPI. Data were expressed as mean (SD). P values were calculated using one-way ANOVA followed by Newman-Keuls test or Students-unpaired *t*-test. ∗P < 0.05, ∗∗P < 0.01,∗∗∗P < 0.001,∗∗∗∗P < 0.0001.

Initially, through bioinformatics analysis of an existing dataset (GSE38974), we identified reduced mRNA levels of arylsulfatase family member K and J in COPD patients (Fig. 1A and B). To validate this finding we collected 18 non-smokers, 18 healthy-smokers and 20 COPD patients with the clinical information shown in Table S1. Compared with non-smokers, ARSK was significantly downregulated in healthy smokers and COPD patients. Whereas, we didn't observe a significant change of ARSJ among these groups (Fig. 1C and D). Interestingly, the mRNA levels of ARSK in COPD patients showed a positive correlation with lung function, as measured by FEV_1_ % predicted (Fig. 1E). Furthermore, we noted that the reduced expression of ARSK was primarily localized to the airway epithelium (Fig. 1F). Consistent with alterations in clinical specimens, we found a similar reduction of Arsk in lung tissues of CS-exposed mice, especially in airway epithelium (Fig. 1G and H). In *vitro*, when HBE cells were exposed to CSE at varying concentrations and durations, a downregulation of ARSK was also evident (Fig. 1I–K).

### 3.2 ARSK mitigated cellular senescence induced by CSE in bronchial epithelial cells

To illustrate the role of ARSK in airway epithelium, we initially conducted an RNA-sequencing analysis on HBE cells transfected with siARSK or control siRNA. After enrichment analysis, these DEGs were found to be associated with biological processes related to cell senescence, including aging, DNA damage response, DNA repair, inflammatory response and regulation of cell cycle (Fig. S1A and B).

**Fig. 2:**
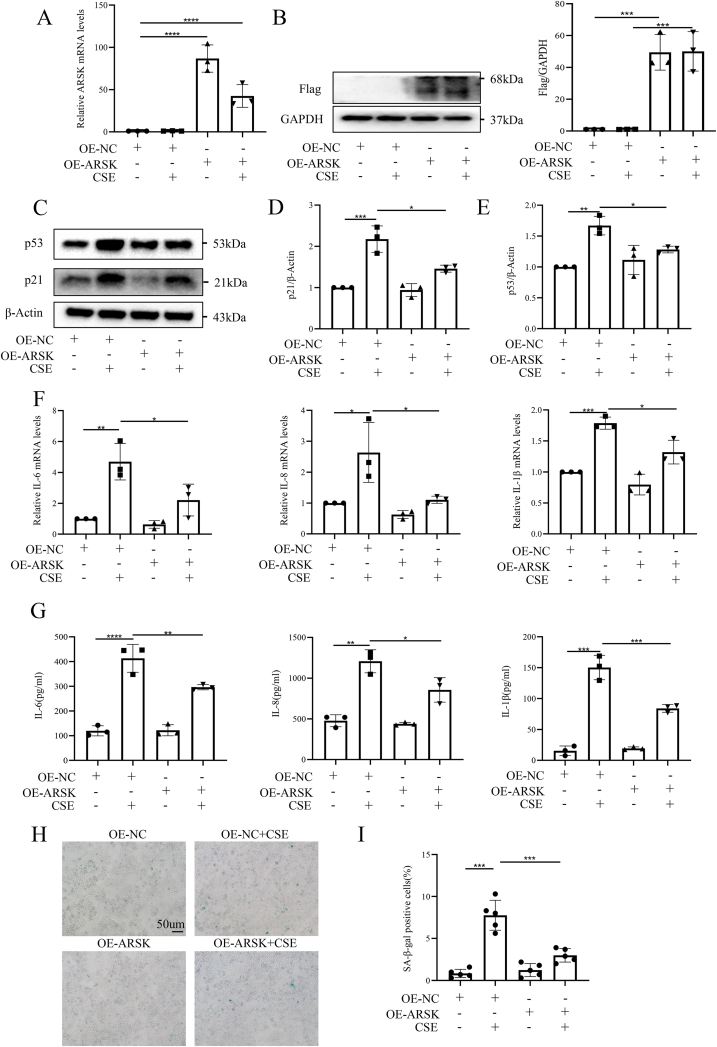
ARSK overexpression attenuated cellular senescence in CSE-induced HBE cells. (**A**) Relative ARSK mRNA levels in HBE cells after ARSK-plasmid transfection. (**B**) Western blot analysis of Flag after the transfection of ARSK plasmid. (**C-E**) Western blot analysis of P21 and P53 expression in CSE(10 %)-induced HBE cells with ARSK overexpression. (**F and G**) The relative mRNA and protein levels of SASP factors in ARSK-overexpressed HBE cells followed by CSE(10 %) treatment. (**H and I**) Representative images of SA-β-gal staining and percentages of SA-β-gal positive cells for HBE cells with ARSK overexpression. Scale bar = 50μm, magnification = 200x. Data were expressed as mean (SD). P values were calculated using one-way ANOVA followed by Newman-Keuls test or Students-unpaired *t*-test. ∗P < 0.05, ∗∗P < 0.01,∗∗∗P < 0.001,∗∗∗∗P < 0.0001; SASP: senescence associated secretory phenotype.

Subsequently, we constructed HBE cells with ARSK overexpression or knockdown to explore its impact on cellular senescence. On one hand, transfection of HBE cells with the plasmid resulted in ARSK overexpression (Fig. 2A and B). Upon CSE stimulation, the upregulation of ARSK significantly reduced the expressions of p21 and p53, two markers of cell senescence (Fig. 2C–E). What's more, overexpressing ARSK downregulated the expression of SASP factors IL-6, IL-8 and IL-1β at mRNA levels, as well as at protein levels, in CSE-treated HBE cells (Fig. 2F and G). Consistently, the results of SA-β-gal staining revealed a lower percentage of positive cells after ARSK overexpression in response to CSE (Fig. 2H and I).

On the other hand, we transfected HBE cells with ARSK siRNA and ensured the knockdown effect (Fig. S2A). As expected, decreased ARSK expression significantly exacerbated the expression of p21 and p53 after CSE treatment, as well as the number of β-gal-positive cells (Fig. S2B–F). Additionally, the reduction of ARSK enhanced expressions and secretions of SASP factors IL-6, IL-8 and IL-1β in CSE-induced HBE cells (Fig. S2G and H). Therefore, it's plausible that ARSK could ameliorate cell senescence in bronchial epithelial cells.

### 3.3 ARSK alleviated CSE-induced excessive mitophagy and mitochondrial dysfunction in bronchial epithelial cells

To clarify the potential mechanisms by which ARSK affects cell senescence, we furtherly analyzed the RNA-sequencing results and found these DEGs were enriched in KEGG pathways associated with autophagy and mitophagy (Fig. S1C). Given the critical role of mitophagy and mitochondrial function in cell senescence, we detected the markers of mitophagy and mitochondrial function in HBE cells after ARSK overexpression or knockdown.

**Fig. 3:**
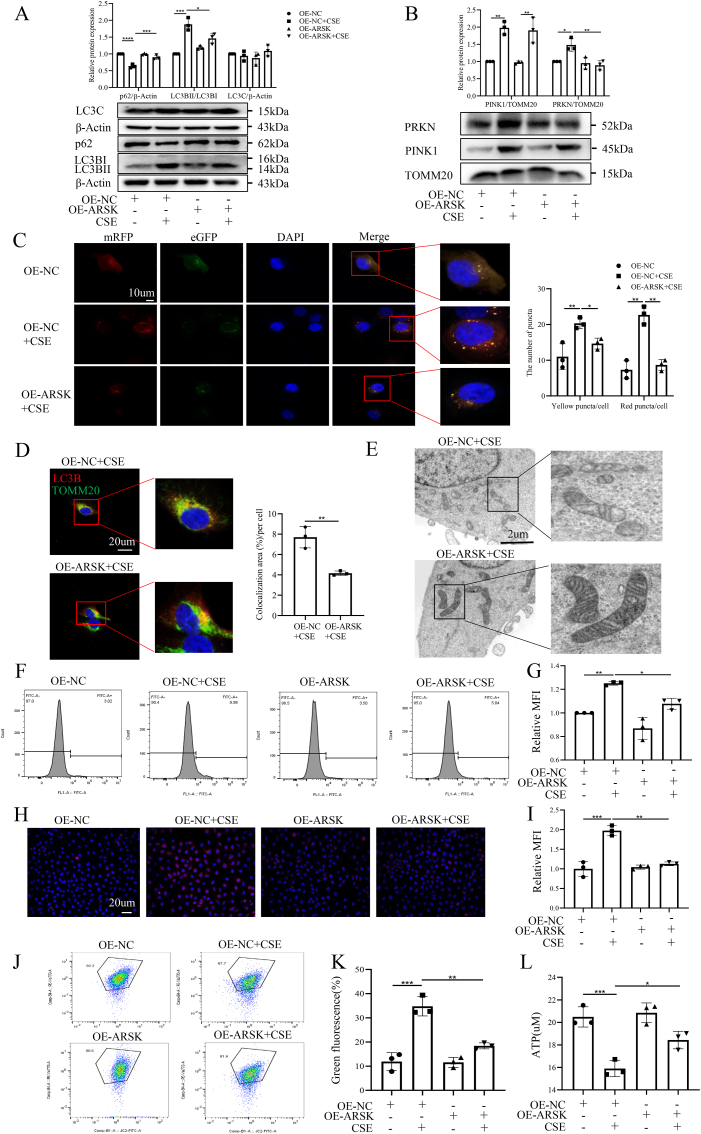
ARSK overexpression could ameliorate CSE-induced excessive mitophagy and mitochondrial dysfunction in HBE cells. (**A and B**) Western blot analysis of mitophagy-related proteins from whole cell lysates or mitochondrial fractions in CSE (10 %)-treated HBE cells after ARSK overexpression. (**C**) Typical confocal images of autophagic flux, along with the quantitative data for yellow and red puncta counts. Scale bar = 10μm,magnification = 1000x. (**D**) Typical confocal images for immunostaining of TOMM20 and LC3B in HBE cells with ARSK overexpression, along with the semiquantitative data of colocalization area. Red color-LC3B, Green color-TOMM20, Blue color-DAPI. Scale bar = 20μm,magnification = 1000x.(**E**)Representative electron microscopic images of CSE (10 %) -stimulated HBE cells with or without ARSK overexpression. Scale bar = 2um. (**F and G**) Representative images of intracellular ROS levels and mean fluorescence intensity for CSE (10 %)-induced HBE cells after ARSK plasmid transfection. (**H and I**) Representative images of Mitosox staining and relative mean fluorescence intensity (MFI) for treated and untreated HBE cells. Red color-Mitosox, Blue color-DAPI. Scale bar = 20μm, magnification = 400x. (**J and K**) The mitochondrial membrane potential of HBE cells measured by flow cytometry and statistical graph of green fluorescence. (**L**) The levels of ATP content in HBE cells under CSE (10 %) treatment after the transfection of ARSK plasmid. Data were expressed as mean (SD). P values were calculated using one-way ANOVA followed by Newman-Keuls test or Students-unpaired *t*-test. ∗P < 0.05, ∗∗P < 0.01,∗∗∗P < 0.001,∗∗∗∗P < 0.0001.

In experiments with ARSK overexpression, Western blot analysis revealed that upregulating ARSK obviously decreased the elevation in the ratios of PRKN/TOMM20 (translocase of outer mitochondrial membrane 20), and LC3BII/LC3BI, respectively increased the expression of p62 in CSE-induced HBE cells. Intriguingly, ARSK upregulation had no impact on the expression of PINK1 in mitochondria following CSE stimulation. Given that LC3C is reported to be implicated in the mitophagy process, we also examined it. However, no significant differences were observed among the groups. (Fig. 3A and B). The detection of autophagic flux revealed a significant rise in yellow and red puncta in HBE cells following CSE stimulation, indicative of the formation of autophagosomes and autolysosomes. However, this effect was notably diminished when ARSK was overexpressed (Fig. 3C). Additionally, the immunostaining results for TOMM20 and LC3B demonstrated that the colocalization area in CSE-stimulated HBE cells was reduced after ARSK overexpression (Fig. 3D). Using electron microscopy, we observed that HBE cells with ARSK overexpression had better morphology of mitochondria after CSE stimulation (Fig. 3E). Accordingly, we observed a significant decrease in the levels of intracellular and mitochondrial ROS (Fig. 3F–I). Consistently, Flow cytometry results showed that ARSK overexpression alleviated CSE-induced loss of MMP (Fig. 3J and K). Furthermore, CSE-induced loss of ATP content was partially reserved by ARSK upregulation (Fig. 3L).

In contrast, ARSK knockdown experiments showed that suppressing ARSK intensified CSE-induced expressions of the aforementioned mitophagy makers except for PINK1 (Fig. S3A–B). Consistent alterations were also observed in the autophagic flux analysis and the immunostaining of TOMM20 and LC3B. (Fig. S3C–D). Under electron microscopy, the mitochondria in ARSK-knockdown HBE cells exhibited more disrupted morphology after CSE stimulation, with a reduced number of cristae. Additionally, the presence of lysosomes around the mitochondria suggested the activation of mitophagy (Fig. S3E). Moreover, suppressing ARSK aggravated the increase of intracellular and mitochondrial ROS levels (Fig. S3F–I), as well as the loss of MMP and ATP in CSE-treated HBE cells (Fig. S3J–L). In general, ARSK could relieve excessive mitophagy and mitochondrial damage induced by CSE, which may be one of mechanisms regulating cell senescence.

### 3.4 ARSK knockdown did not significantly impact lysosomal function in CSE-stimulated HBE cells, at least not in terms of the fusion of autophagosomes with lysosomes

As ARSK is a member of lysosomal enzymes, we sought to investigate whether ARSK knockdown would impact lysosomal function. Our results demonstrated that reduction of ARSK further enhanced CSE-induced lysosomal biogenesis. However, it did not affect the expression of galectin-3, a marker of lysosomal membrane damage (Fig. S4A and B). To further assess lysosomal integrity, we performed an AO staining assay, which revealed that ARSK downregulation had no significant impact on lysosomal integrity (Fig. S4C and D). Additionally, the lysotracker and lysosensor staining assays was conducted. The results showed that while CSE treatment promoted lysosomal acidity, ARSK knockdown had no obvious influence on it (Fig. S4E and F). Moreover, the colocalization of LAMP1 and LC3B indicated that the fusion between autophagosomes and lysosomes was enhanced following the combination of ARSK knockdown and CSE stimulation (Fig. S4G and H). In assessing lysosomal proteolytic activity, we detected the expression of Cathepsin B (CTSB). Our results indicated that ARSK knockdown didn't impair CSE-stimulated CTSB upregulation (Fig. S4I and J). In summary, the decrease of ARSK appears to have minimal impact on lysosomal function in CSE-treated HBE cells, specifically in terms of the fusion between autophagosomes and lysosomes.

### 3.5 Mitophagy mediated the regulatory effects of ARSK on cell senescence

**Fig. 4:**
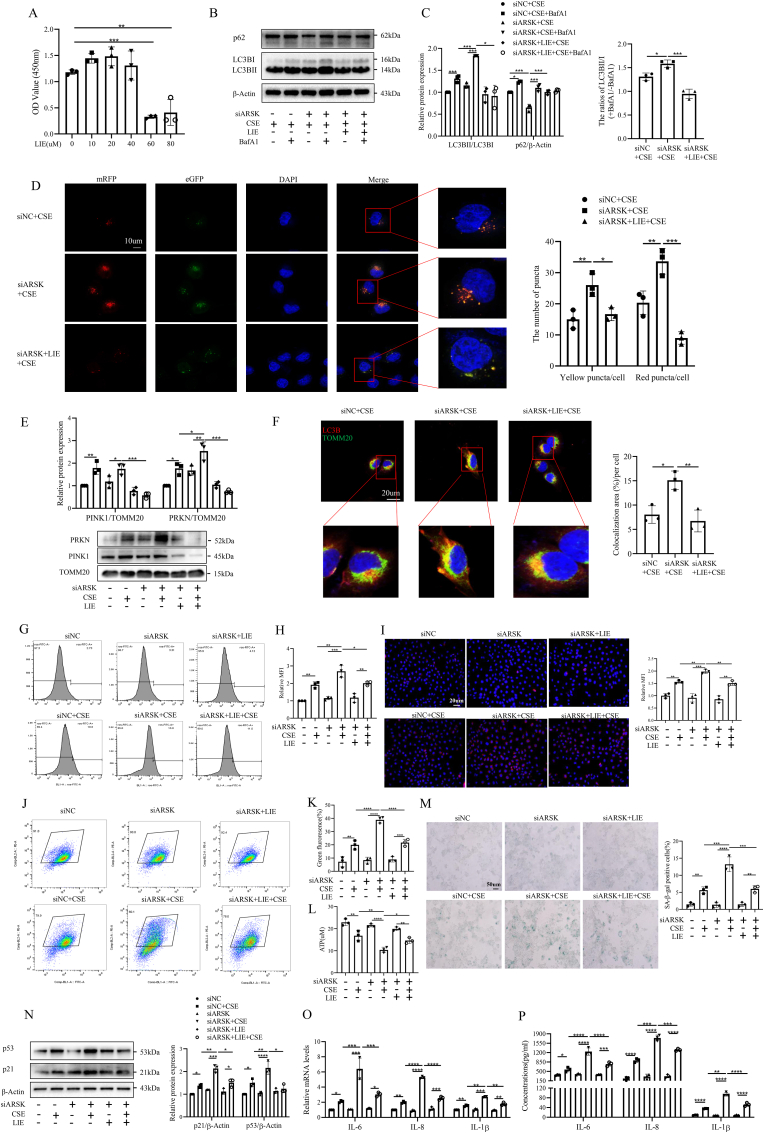
The detrimental effects of ARSK knockdown on mitochondrial dysfunction and cellular senescence was partly reserved by mitophagy inhibitor Liensinine. (**A**) The viability of HBE cells treated with different concentration of Liensinine(LIE) measured by CCK8 assay. (**B and C**) Western blot analysis of LC3B and p62 for whole cell lysates from HBE cells after ARSK knockdown with or without BafA1 treatment. (**D**) Typical confocal images of autophagic flux, along with quantitative data of yellow and red puncta counts. Scale bar = 10μm,magnification = 1000x. (**E**) Western blot analysis of PINK1 and PRKN for mitochondrial proteins in HBE cells. (**F**) Confocal images of Immunostaining for TOMM20 and LC3B in HBE cells with ARSK knockdown followed by LIE and CSE(10 %) treatment, with relative semiquantitative result. Red color-LC3B, Green color-TOMM20, Blue color-DAPI. Scale bar = 20μm, magnification = 1000x. (**G and H**) Representative images of intracellular ROS levels and mean fluorescence intensity for HBE cells in each group. (**I**) Representative images of Mitosox staining for treated and untreated HBE cells. Red color-Mitosox, Blue color-DAPI. Scale bar = 20μm, magnification = 400x. (**J and K**) The mitochondrial membrane potential of HBE cells measured by flow cytometry and statistical graph of percentage for green fluorescence. (**L**) The levels of ATP content in HBE cells with ARSK knockdown followed by LIE and CSE(10 %) treatment. (**M**) Representative images of SA-β-gal staining and percentages of SA-β-gal positive cells for HBE cells in each group. Scale bar = 50μm, magnification = 200x. (**N**)Western blot analysis of p21, p53 for whole cell lysates from LIE-treated HBE cells after ARSK knockdown. (**O and P**) The relative mRNA and protein levels of SASP factors in HBE cells treated by LIE and CSE(10 %) after knockdown of ARSK. Data were expressed as mean (SD). P values were calculated using one-way ANOVA followed by Newman-Keuls test or Students-unpaired *t*-test. ∗P < 0.05, ∗∗P < 0.01,∗∗∗P < 0.001,∗∗∗∗P < 0.0001; SASP: senescence associated secretory phenotype.

To further explore if the regulatory effects of ARSK on cell senescence were mediated by mitophagy, we treated HBE cells with the mitophagy inhibitor Liensinine (LIE) following ARSK knockdown. The CCK8 assay results suggested a propriate cell viability at the concentration of 40uM (Fig. 4A). Subsequently, we treated parallel cell groups with BafA1(100 nM,12h) and the result demonstrated that the inhibition of LC3BII expression in HBE cells following ARSK knockdown was attributed to a decrease in autophagic flux, rather than compromised lysosomal degradation. Moreover, it was observed that LIE treatment effectively suppressed autophagy (Fig. 4B and C). Confocal microscopy further confirmed this inhibition of autophagy, with a notable reduction in yellow and red puncta (Fig. 4D). Additionally, the decreased expression of PRKN and reduced colocalization of TOMM20 and LC3B further validated the inhibition of mitophagy after LIE treatment (Fig. 4E and F). Notably, LIE improved mitochondrial damage induced by ARSK knockdown, as evidenced by the reductions in intracellular and mitochondrial ROS levels (Fig. 4G–I), and the preservations of MMP and ATP content (Fig. 4J–L). Lastly, SA-β-gal staining results demonstrated that LIE treatment obviously decreased the percentage of positive cells (Fig. 4M). Consistent with the change, the expressions of p21, p53 and SASP factors IL-6, IL-8 and IL-1β were both relived in HBE cells by LIE (Fig. 4N–P). Generally, these data suggested that ARSK regulated cell senescence through mitophagy in bronchial epithelial cells.

### 3.6 ARSK relieved CSE-induced mitophagy and cell senescence via regulating the phosphorylation of PRKN at serine 65

**Fig. 5:**
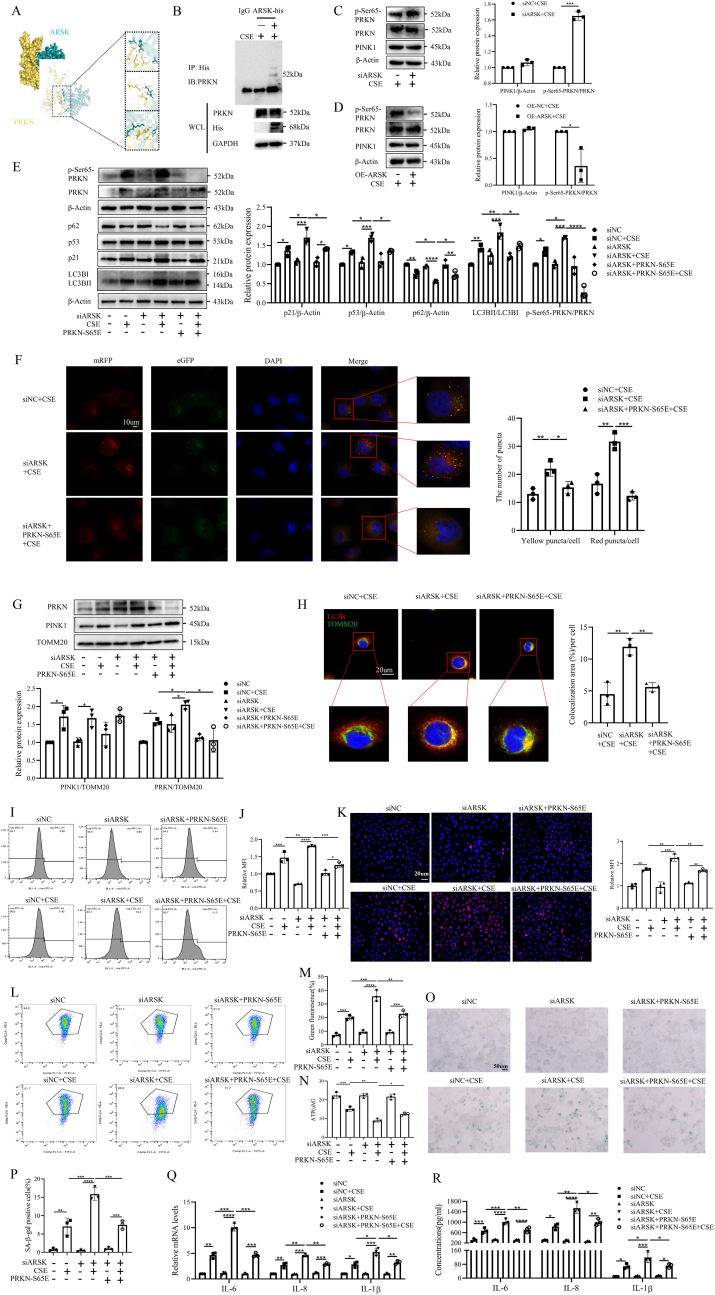
ARSK alleviated mitochondrial dysfunction and cellular senescence through inhibiting the phosphorylation of Parkin(PRKN) at serine 65 in HBE cells. (**A**) 3D structure of protein-protein docking model for ARSK and PRKN. (**B**) Co-immunoprecipitation analysis of ARSK and PRKN. (**C and D**) Western blot analysis of *p*-Ser65-PRKN, PINK1 and PRKN in HBE cells after ARSK knockdown or overexpression. (**E**) Western blot analysis of p21, p53 and mitophagy-related proteins in whole cell lysates of HBE cells with knockdown of ARSK combined with PRKN mutation. (**F**) Representative confocal images for HBE cells with infection of mRFP-eGFP-LC3B adenovirus, with quantitative data of yellow and red puncta counts. Scale bar = 10μm,magnification = 1000x. (**G**) Western blot analysis of PINK1 and PRKN in mitochondrial proteins of HBE cells. (**H**) Confocal images for TOMM20 and LC3B in HBE cells with knockdown of ARSK and PRKN mutation followed by CSE(10 %) treatment, with semiquantitative result. Red color-LC3B, Green color-TOMM20, Blue color-DAPI. Scale bar = 20μm, magnification = 1000x. (**I and J**) Representative images of intracellular ROS levels and mean fluorescence intensity for HBE cells with or without transfection of siARSK and PRKN S65E plasmid. (**K**) Representative images of Mitosox staining for treated and untreated HBE cells. Red color-Mitosox, Blue color-DAPI. Scale bar = 20μm, magnification = 400x. (**L and M**) The mitochondrial membrane potential of HBE cells measured by flow cytometry and statistical graph of percentage for green fluorescence. (**N**) The levels of ATP content in HBE cells under different conditions followed by CSE(10 %) treatment. (**O and P**) Representative images of SA-β-gal staining and percentages of SA-β-gal positive cells for HBE cells in each group. Scale bar = 50μm, magnification = 200x. (**Q and R**) The relative mRNA and protein levels of SASP factors for CSE(10 %)-treated HBE cells after knockdown of ARSK combined with PRKN mutation. Data were expressed as mean (SD). P values were calculated using one-way ANOVA followed by Newman-Keuls test or Students-unpaired *t*-test. ∗P < 0.05, ∗∗P < 0.01,∗∗∗P < 0.001,∗∗∗∗P < 0.0001; SASP: senescence associated secretory phenotype; WCL: whole cell lysates.

Based on that ARSK affected the expression of PRKN but not PINK1 in mitochondria, and the importance of PRKN as an initiator of mitophagy, we hypothesized an interaction between ARSK and PRKN. We initiated our investigation with protein-protein docking, which predicted numerous hydrogen bonds and salt bridges between ARSK and PRKN (Fig. 5A). This prediction was substantiated by Co-IP experiments, confirming their interaction in HBE cells. Interestingly, we observed that PRKN in the whole-cell lysate showed no significant changes, which was contradictory to the alterations observed in mitochondrial proteins after ARSK overexpression (Fig. 5B). We speculated that this result might be due to the interaction between ARSK and PRKN, which affects the translocation of PRKN to the mitochondria. As is widely recognized, PRKN must be phosphorylated at serine 65 to become activated and subsequently translocated to the mitochondria to exert its function [15]. Therefore, we subsequently examined the expression levels of *p*-Ser65-PRKN in whole cell lysate and found that its expression, but not PRKN, significantly increased upon ARSK knockdown and vice versa (Fig. 5C and D). To further determine whether ARSK affects the cellular senescence by regulating the phosphorylation of PRKN, we constructed a point mutation plasmid of PRKN at the serine 65 site (PRKN S65E). The results of Western blot from the whole cell lysate indicated the PRKN S65E partially reversed the upregulations of p62, LC3BII/LC3BI, p21 and p53 caused by ARSK knockdown (Fig. 5E). Furthermore, autophagic flux assays demonstrated that PRKN S65E effectively suppressed autophagy (Fig. 5F). Additionally, Western blot of mitochondrial proteins revealed that PRKN S65E relived the translocation of PRKN to mitochondria after ARSK knockdown (Fig. 5G). Confocal images also showed the decreased mitophagy owing to the transfection of PRKN S65E plasmid (Fig. 5H). As before, we detected intracellular and mitochondrial ROS, MMP and ATP content. Similarly, the results displayed that HBE cells treated with ARSK knockdown in combination with PRKN mutation exhibited improved mitochondrial function compared to those with only ARSK knockdown after CSE treatment (Fig. 5I–N). Finally, we evaluated the level of cell senescence using SA-β-gal staining, qPCR and ELISA assays. Consistently, we found that ARSK-induced cell senescence was relieved by point mutation of PRKN in HBE cells (Fig. 5O–R). In conclusion, it seemed like ARSK interacted with PRKN to influence the phosphorylation of PRKN at serine 65, thereby alleviating cellular senescence.

### 3.7 Intratracheal administration of AAV2/9-Arsk alleviated lung destruction, mitophagy and epithelial cellular senescence induced by cigarette smoke

**Fig. 6:**
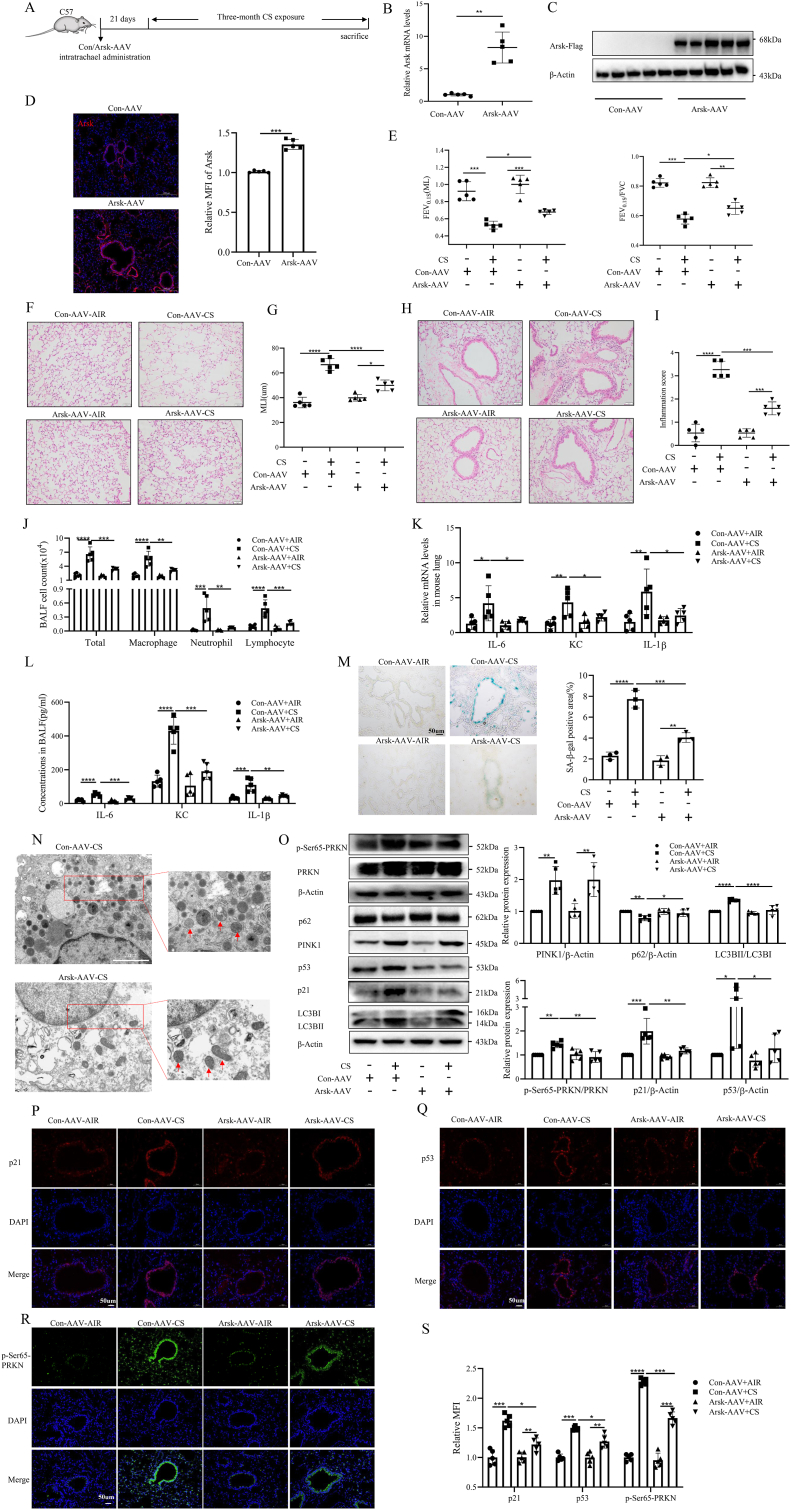
ARSK overexpression prevented mice from CS-induced lung destruction, mitophagy and epithelial cellular senescence. (**A**) The schematic diagram of Control/Arsk-AAV airway administration and CS-exposure schedule. (**B and C**) Confirmation of Arsk overexpression in lung tissues of Arsk-AAV mice. (**D**) Representative FISH images and quantitative data of MFI for Arsk in mouse lung sections. Scale bars = 200μm, magnification = 200x, Red color-Arsk, Blue color-DAPI. (**E**) The results of lung function. (**F and G**) Representative images of HE staining of mouse lung tissues and the results of mean linear intercept(MLI) to evaluate lung emphysema. Scale bar = 50μm, magnification = 200x. (**H and I**) Representative images of HE-stained lung paraffin sections and semiquantitative result of inflammation score to evaluate airway inflammation. Scale bar = 50μm, magnification = 200x. (**J**) Numbers of total cells, macrophages, neutrophils and lymphocytes in mouse bronchoalveolar lavage fluid(BALF). (**K**) Relative mRNA Levels of SASP factors in mouse lung tissues. (**L**) Levels of SASP factors in mouse BALF measured by ELISA. (**M**) SA-β-gal staining of frozen lung sections, along with quantitative result. Scale bar = 50μm, magnification = 200x. (**N**) Transmission electron microscopic images for mitochondria in bronchial epithelial cells of CS-exposed mice. The red arrowheads represent mitochondria. Scale bar = 2um. (**O**) Western blot results of the expression of p21, p53 and mitophagy-related proteins in mouse lung tissues. (**P–S**) Representative images of immunostaining of p21, p53 and *p*-Ser65-PRKN in mouse lung tissues and semiquantitative results of relative mean fluorescence intensity (MFI). Scale bar = 50μm, magnification = 200x. Data were expressed as mean (SD). P values were calculated using one-way ANOVA followed by Newman-Keuls test or Students-unpaired *t*-test. ∗P < 0.05, ∗∗P < 0.01,∗∗∗P < 0.001,∗∗∗∗P < 0.0001. FEV_0.1s_: forced expiratory volume in 0.1s; FVC: forced vital capacity; SASP: senescence associated secretory phenotype.

To elucidate the therapeutic potential of ARSK in COPD, we overexpressed Arsk in the lungs of mice by intratracheal administration of AAV2/9-Arsk. 21 days post-administration, the mice were subjected to air or CS for 3 months (Fig. 6A). Lung Arsk levels remained elevated significantly in Arsk-AAV mice contrasting to Con-AAV mice after 3-month CS exposure (Fig. 6B and C). Additionally, FISH staining revealed that the up-regulation of ARSK expression was particularly prominent in the airway epithelium (Fig. 6D). Arsk-AAV mice exhibited a less pronounced decline in lung function than Con-AAV mice after CS exposure (Fig. 6E). HE staining of mouse lung tissues also revealed CS-exposed Arsk-AAV mice had milder emphysema (Fig. 6F and G). In terms of lung inflammation, also known as a part of lung aging, CS-exposed Arsk-AAV mice showed reduced airway inflammation, lower counts of total cells, macrophages, neutrophils and lymphocytes in BALF, as well as significantly decreased expressions and secretions of IL-6, KC and IL-1β in lung tissues compared to CS-exposed Con-AAV mice (Fig. 6H–L). Additionally, SA-β-gal staining of frozen lung sections indicated a lower level of airway epithelial senescence in CS-exposed Arsk-AAV mice (Fig. 6M). Through transmission electron microscopy, we observed a better condition of mitochondria in airway epithelial cells of CS-exposed Arsk-AAV mice compared to Con-AAV mice (Fig. 6N). Next, Western blot results confirmed significantly downregulated expressions of mitophagy markers, p21 and p53 (Fig. 6O). Consistently, p21, p53 and *p*-Ser65-PRKN expressions were lower in airway epithelial cells of Arsk-AAV mice compared to Con-AAV mice after CS exposure (Fig. 6P–S). Overall, ARSK overexpression in the lung could effectively improve lung function, mitigate mitophagy and reduce epithelial cellular senescence in CS-exposed mice.

### 3.8 LIE treatment ameliorated lung destruction, mitophagy and epithelial cellular senescence in CS-exposed ARSK AEC-KO mice

**Fig. 7:**
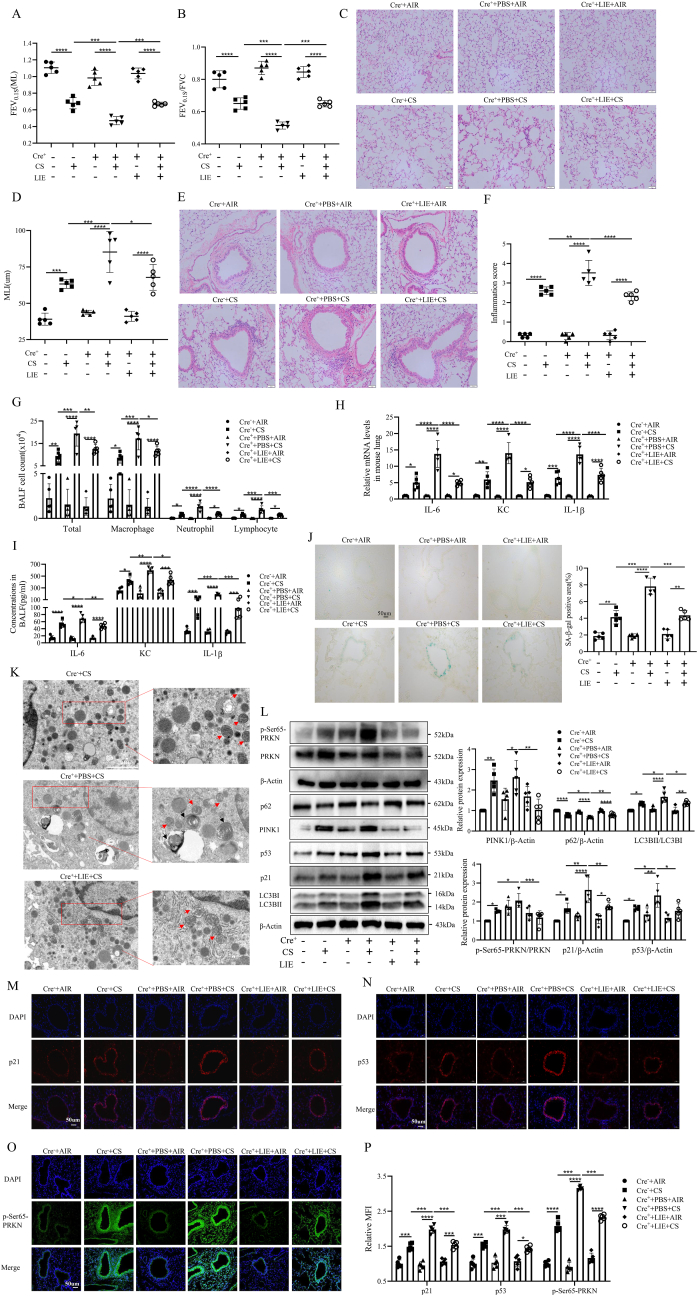
Liensinine treatment could ameliorate CS-induced lung pathology, mitophagy and epithelial cellular senescence in Arsk AEC-KO mice. (**A and B**) The results of lung function. (**C and D**) Representative images of HE staining of mouse lung tissues and results of MLI. Scale bar = 50μm, magnification = 200x. (**E and F**) Representative images of HE-stained lung paraffin sections and semiquantitative result of inflammation score. Scale bar = 50μm, magnification = 200x. (**G**) Numbers of total cells, macrophages, neutrophils and lymphocytes in BALF of mouse. (**H**) mRNA levels of SASP factors in lung tissues of mouse. (**I**) ELISA results of SASP factors in mouse BALF. (**J**) SA-β-gal staining of frozen lung sections, along with relative quantitative result. Scale bar = 50μm, magnification = 200x. (**K**) Transmission electron microscopy for detection of mitochondria in bronchial epithelial cells of CS-exposed mice. The red arrowheads represent mitochondria, the black arrowhead represent autolysosomes. Scale bar = 2um. (**L**) results of the expression of p21, p53 and mitophagy-related proteins measured by Western blot. (**M-P**) Representative images of immunostaining of p21,p53 and *p*-Ser65-PRKN in mouse lung tissues and semiquantitative result of relative mean fluorescence intensity (MFI). Scale bar = 50μm, magnification = 200x. Data were expressed as mean (SD). P values were calculated using one-way ANOVA followed by Newman-Keuls test or Students-unpaired *t*-test. ∗P < 0.05, ∗∗P < 0.01,∗∗∗P < 0.001,∗∗∗∗P < 0.0001. AEC: airway epithelial cell; FEV_0.1s_: forced expiratory volume in 0.1s; FVC: forced vital capacity. SASP: senescence associated secretory phenotype.

In this part, we first generated mice with specific knockout of Arsk in airway epithelial cells (AEC) using the CRISPR-Cas9 system (Fig. S5A). Following exposure to CS for three months, these mice received daily oral administration of LIE at the concentration of 5 mg/kg or PBS for the final month (Fig. S5B). Genotyping confirmed the presence of the flox allele from ARSK^flox/flox^, WT and the Scgb1a1-Cre allele, with a significant reduction of Arsk observed in lung tissues of Cre^+^ mice compared with Cre^−^ mice (Fig. S5C and D). Moreover, LIE treatment didn't significantly affect the structural integrity of heart, liver, spleen and kidney tissues in mice (Fig. S5E). However, Cre^+^ mice exhibited a stronger decline of lung function than Cre^−^ mice which was reserved by LIE-treatment after CS exposure (Fig. 7A and B). HE staining of lung tissues also revealed that CS-exposed Cre^+^ mice had the most severe emphysematous changes and airway inflammation among these groups (Fig. 7C–F). Similarly, CS-exposed Cre^+^ mice had the highest counts of total cells, macrophages, neutrophils and lymphocytes in BALF (Fig. 7G). Regarding SASP factors, there were highest expressions and secretions of IL-6, KC and IL-1β in lung tissues of Cre^+^ mice after CS exposure (Fig. 7H and I). We could also discover that LIE treatment ameliorated CS-induced airway epithelial cellular senescence and mitochondrial damage in Cre^+^ mice (Fig. 7J and K). Western blot analysis of mouse lung proteins showed consistent trends in the expressions of mitophagy and senescence markers (Fig. 7L). Finally, we confirmed the highest expressions of p21, p53 and *p*-Ser65-PRKN in airway epithelial cells of CS-exposed Cre^+^ mice using immunofluorescence (Fig. 7M–P). Therefore, similar to *in vitro* observations, LIE treatment could also alleviate mitophagy and cell senescence induced by ARSK deficiency *in vivo*.

### 3.9 Lower expression of ARSK in response to CS exposure was mediated by transcriptional regulation of AR

**Fig. 8:**
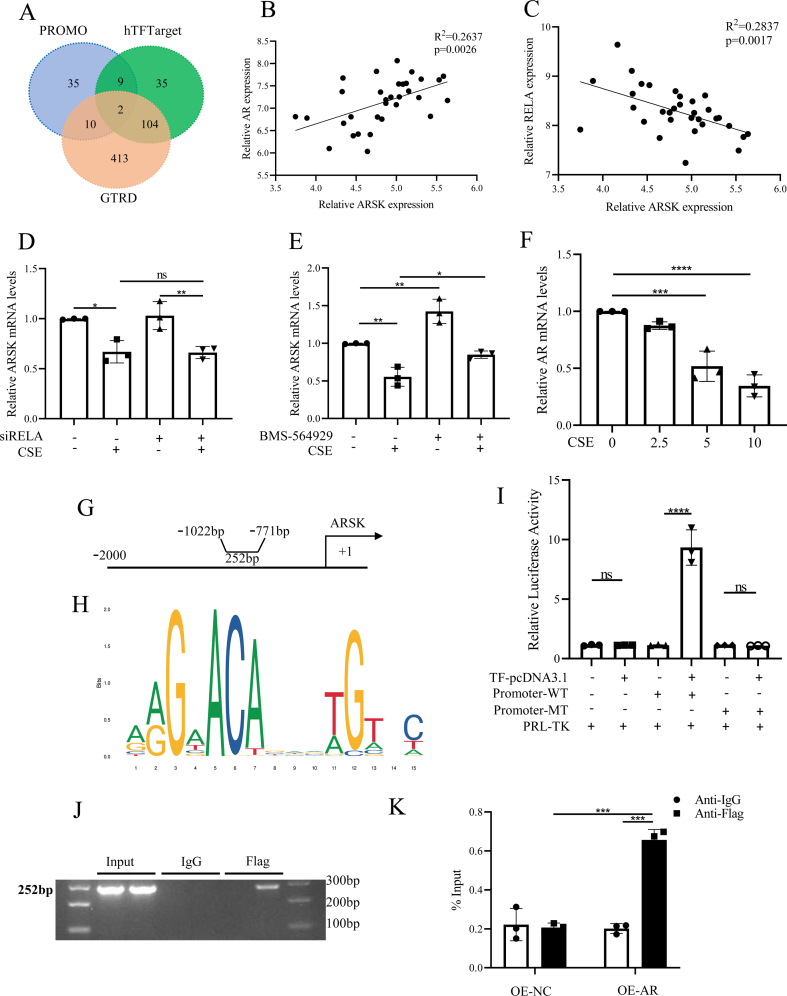
Lower expression of ARSK after CSE treatment was mediated by transcriptional regulation of AR. (**A**) The Venn plot of predicted transcriptional factors in the promoter region of ARSK from 3 databases. (**B**) The correlation analysis of relative ARSK and AR expressions in GSE38974. (**C**) The correlation analysis of relative ARSK and RELA expressions in GSE38974. (**D**) Relative ARSK mRNA levels with or without the transfection of RELA in HBE cells. (**E**) Relative ARSK mRNA levels with or without the treatment of BMS-564929 (AR agonist) in HBE cells. (**F**) Relative AR mRNA levels after CSE treatment in HBE cells. (**G**) Schematic diagram of luciferase reporter gene constructs with ARSK promoter. (**H**) The sequence logo of transcription factor AR. (**I**) Relative luciferase activity in HBE cells with ARSK wildtype or mutant promoter with AR pcDNA3.1. (**J and K**) ChIP assay showed AR could bind to ARSK in HBE cells. Data were expressed as mean (SD). P values were calculated using one-way ANOVA followed by Newman-Keuls test. ∗P < 0.05, ∗∗P < 0.01, ∗∗∗P < 0.001, ∗∗∗∗P < 0.0001.

To explore the suppression of ARSK expression after CSE treatment, we predicted potential transcription factors for ARSK utilizing 3 databases PROMO (https://alggen.lsi.upc.edu/recerca/menu_recerca.html), hTFtarget (https://guolab.wchscu.cn/hTFtarget/#!/) and GTRD (https://gtrd20-06.biouml.org/). The intersection of these databases yielded two candidate factors (Fig. 8A). Correlation analysis revealed a positive correlation between ARSK and AR expressions, and a negative correlation with RELA expressions (Fig. 8B and C). Subsequently, we transfected HBE cells with siRNA targeting RELA to verify its regulation on ARSK. However, the result suggested the knockdown of RELA seemed no significant influence on ARSK expression (Fig. 8D). In contrast, treatment of HBE cells with the AR agonist BMS-564929 demonstrated a positive regulatory effect on ARSK (Fig. 8E). What's more, we found an obvious decrease in AR expression after CSE treatment (Fig. 8F). To furtherly confirm that AR could bind to the promoter of ARSK, we first acquired the promoter sequence of ARSK from NCBI and identified AR response elements (Fig. 8G and H). Luciferase activity assays confirmed the binding of AR to the wildtype ARSK promoter, but not to the mutant promoter (Fig. 8I). ChIP PCR analysis using AR-Flag-conjugated DNA samples showed amplifications of ARSK primers (Fig. 8J and K). In conclusion, the downregulation of ARSK in HBE cells after CSE treatment appeared to be mediated by transcriptional regulation of AR.

## 4 Discussion

In our study, we identified the decreased expression of ARSK in COPD patients and COPD model mice. We also confirmed a protective role of ARSK in the airway epithelial cellular senescence of COPD *in vivo* and *in vitro*. Mechanistically, ARSK alleviated mitophagy through interaction with PRKN, thus improving mitochondrial dysfunction and subsequent cellular senescence.

In this study, we mainly detected the role of ARSK in mitophagy and cellular senescence of COPD. However, ARSK, as a member of the lysosomal sulfatases family, has been shown to exhibit glucuronate-2-sulfatase activity, which is essential for the degradation of glycosaminoglycans (GAGs) including heparan sulfate (HS), chondroitin sulfate and dermatan sulfate (DS). It has been previously reported that ARSK deficiency can lead to a mild form of mucopolysaccharidosis (MPS) in mice due to GAGs accumulation, with no significant lysosomal storage pathology [26]. Notably, elevated levels of these GAGs, which also contributes to airway inflammation and remodeling, have been detected in the bronchoalveolar lavage (BAL) of COPD patients during exacerbations [27]. Given these observations, ARSK deficiency may also play a role in accumulation of these GAGs and airway remodeling in COPD, a potential role that warrants further investigation. Additionally, to investigate whether the decrease of ARSK would affect lysosomal function and subsequently influence the fusion of autophagosomes with lysosomes, the experiments about lysosomal function were employed. We found that ARSK reduction further increased lysosomal biogenesis but did not affect lysosomal membrane integrity, lysosomal acidity and lysosomal proteolytic activity following CS stimulation. The results of the colocalization of LAMP1 and LC3B also suggested that ARSK downregulation further promoted the formation of autolysosomes. Based on these findings, we concluded that the decrease of ARSK did not significantly affect lysosomal function, particularly with regard to the fusion of autophagosomes with lysosomes.

With a sharp rise in the aging population, in-mounting researches focus on the role of lung aging in age-related disease, particularly COPD, characterized by accelerated lung aging [28]. COPD-associated pathologic mechanisms encompass multiple aging pathways such as cell senescence, stem cell exhaustion, chronic inflammation and epigenetic alterations [29]. Cellular senescence, particularly in the airway epithelium, is marked by cell cycle arrest triggered by the activation of p53 and p21. What's more, senescent cells secret SASP, not only perpetuates chronic inflammation but also promotes further senescence in COPD [30,31]. Senescent cells also exhibit impaired abilities to respond to injuries, potentially leading to abnormal tissue remodeling [32]. The hallmarks of cell senescence include morphological alterations, DNA damage, mitochondrial dysfunction and so on [33]. Not only as a consequence of cellular senescence, mitochondrial dysfunction is also a cause of senescence, creating a vicious cycle [34]. Mitophagy is a process that prevents the accumulation of damaged mitochondria, thus keeping a healthy mitochondrial homeostasis [35]. Although lacking a consensus, mild or adaptive mitophagy is regarded beneficial in alleviating pathologies. In the contrary, excessive or maladaptive mitophagy is defined as a type that exacerbates pathologies [36]. In COPD, the role of mitophagy is controversial. Several studies have demonstrated that cigarette smoke (CS) can induce excessive mitophagy, and inhibiting this process can protect against CS-induced mitochondrial dysfunction [37,38]. For instance, a previous work revealed that PINK1/PRKN pathway upregulated in COPD rat model and in alveolar epithelial cells exposed by CS and PM_2.5_ [39]. Elevated levels of PINK1 and PRKN, which trigger amplified mitophagy, have been identified as a significant contributing factor in the development of COPD [40,41]. Our study echoed these findings, showing a protective role of PRKN inhibition in COPD. Contrasting with this, it's suggested that CS-induced downregulated PRKN could enhance mitochondrial dysfunction and oxidative stress *in vivo* and *in vitro* [16,42]. Additionally, the upregulation of PINK1 and PRKN has been shown to reduce inflammation induced by CS [43]. Interestingly, another study has shown enhanced mitophagy alongside reduced PINK1 and PRKN expression in the lung tissue of CS-exposed mice [44]. The inconsistent role of mitophagy in COPD reflects the complex interplay between mitochondrial dynamics, cellular stress responses and disease progression. In this context, we propose that the varying effects of mitophagy in COPD are likely influenced by factors such as disease stage, dose and duration of CS exposure, cellular heterogeneity, and interactions with other pathological mechanisms. Nevertheless, these findings all underscored that adaptive mitophagy was indispensable to mitochondrial function and the progression of COPD.

The relationship between mitophagy and cellular senescence is also a subject of debate. Generally, senescent cells present impaired mitophagy [45]. However, it was reported that mitophagy, as a part of mitochondrial quality control (MQC) system, might be over-activated in aging due to the accumulation of misfolded proteins and aggregates [46,47]. A comparative study showed that reducing mitophagy activity could be a promising method to impede the senescent state in cardiomyocytes induced by advanced glycation end products [48]. Similarly, excessive degradation by PINK1/PRKN-mediated mitophagy under continuous compression has been shown to accelerate the senescence of nucleus pulposus cells [49]. What's more, Zhou et al. found that enhanced autophagy did not contribute to extending lifespan when the mitochondrial permeability was increased [50]. Murley et al. showed that excessive autophagy in quiescent cells could induce lysosomal damage, leading to impaired cell reactivation with aging [51]. Consistently, in this study, we found that excessive mitophagy exacerbated CS-induced cellular senescence, particularly with the deficiency of ARSK.

In the research about mitophagy, PINK1/PRKN-mediated pathway is one of the most classical routes [52]. Under normal physiological conditions, PINK1 is transported to the mitochondrial inner membrane, where it is rapidly cleaved and degraded. However, when mitochondria are damaged, PINK1 is unable to enter the mitochondrial inner membrane. Instead, it accumulates on the mitochondrial outer membrane. This accumulated PINK1 phosphorylates PRKN and ubiquitin at serine 65, promoting its activation and translocation from the cytosol to the mitochondria [53]. This phosphorylation and translocation of PRKN are the core mechanisms underlying PINK1/PRKN-mediated mitophagy [54]. The phosphorylation and translocation of PRKN could be regulated by multiple factors. For instance, the phosphatase and tensin homolog (PTEN)-long (PTEN-L) is capable of effectively reducing PRKN phosphorylation, thus preventing its mitochondrial translocation [55]. Additionally, Ca^2+^ phosphatase Calcineurin has been suggested to regulate PRKN mitochondrial translocation by direct interactions [54]. In our study, we revealed that ARSK could bind to PRKN and modulate its phosphorylation, thereby affecting its translocation to mitochondria. However, the specific mechanism underlying the interaction between ARSK and PRKN remains to be elucidated.

For the upstream mechanism underlying decreased expression of ARSK after CS exposure, we illuminated androgen receptor (AR) as a transcription factor that regulated the expression of ARSK. Previous studies have reported lower circulating levels of androgen in COPD patients and the beneficial effects of selective androgen receptor modulators on muscle weakness in COPD [56,57]. The sex differences in COPD have been noted since 2007 [58,59], but there's still no definitive explanation as to whether the differences attribute to inherited factors or sex hormones. Our study suggested that CS-induced downregulation of androgen receptor could be involved in the pathogenesis of COPD, opening up numerous intriguing mechanisms that warrant further investigation.

For the limitations in our study, firstly, we were unable to detect the protein levels of ARSK *in vivo* and *in vitro* owing to the unavailability of a specific ARSK antibody. Secondly, we identified the interaction between ARSK and PRKN, but how this interaction regulated the phosphorylation of PRKN at serine 65 remained an open question. Lastly, there are no commercial recombinant proteins or agonists of ARSK, making it impossible to conduct pharmacological intervention experiments in animal models.

In conclusion, our study demonstrated that ARSK downregulation in COPD was mediated by CS-induced decline of AR. We also found that ARSK ameliorated cellular senescence in airway epithelial cells through the modulation of mitophagy via the phosphorylation of PRKN at serine 65. These findings not only advanced our understanding of the molecular mechanisms involved in COPD but also provided a new potential target for therapeutic interventions in COPD.

## CRediT authorship contribution statement

**Ruonan Yang:** Data curation, Investigation, Visualization, Writing – original draft. **Yuan Zhan:** Data curation, Investigation. **Zhesong Deng:** Data curation, Investigation. **Jiaheng Zhang:** Data curation, Investigation. **Shanshan Chen:** Data curation, Investigation. **Yating Zhang:** Data curation, Investigation. **Hao Fu:** Data curation, Investigation. **Xiangling Meng:** Data curation, Investigation. **Jixing Wu:** Investigation, Methodology, Visualization. **Yiya Gu:** Investigation, Methodology, Visualization. **Qian Huang:** Investigation, Methodology, Visualization. **Congyi Wang:** Investigation, Methodology, Visualization. **Jungang Xie:** Funding acquisition, Supervision, Writing – review & editing.

## Materials availability

This study did not generate new unique reagents.

## Data and code availability

Any additional information required to reanalyze the data reported in this paper is available from the lead contact, Jungang Xie (xiejjgg@hotmail.com) upon request. This study does not include original code.

## Financial support

This study was supported by the 10.13039/501100001809National Natural Science Foundation of China (No. 82170049, 81973986), the Leading talents of public health in Hubei Province (2022SCZ047), the Clinical Collaboration Project of Traditional Chinese and 10.13039/100007159Western Medicine in the Major Difficult Diseases in Hubei Province (Respiratory system Diseases), the Project of Key R&D Program in Hubei Province (2023BCB127) and Noncommunicable Chronic Diseases-10.13039/501100018537National Science and Technology Major Project (2023ZD0506300 and 2024ZD0528400).

## Declaration of competing interest

The authors declare that they have no known competing financial interests or personal relationships that could have appeared to influence the work reported in this paper.
