# Supplementary material for: Arylsulfatase K attenuates airway epithelial cell senescence in COPD by regulating parkin-mediated mitophagy
Source: Redox Biol. 2025 Jul 31;86:103793. doi: 10.1016/j.redox.2025.103793 (PMC12344987; doi:10.1016/j.redox.2025.103793)
Supplement: Multimedia component 1 [file mmc1.docx]

**
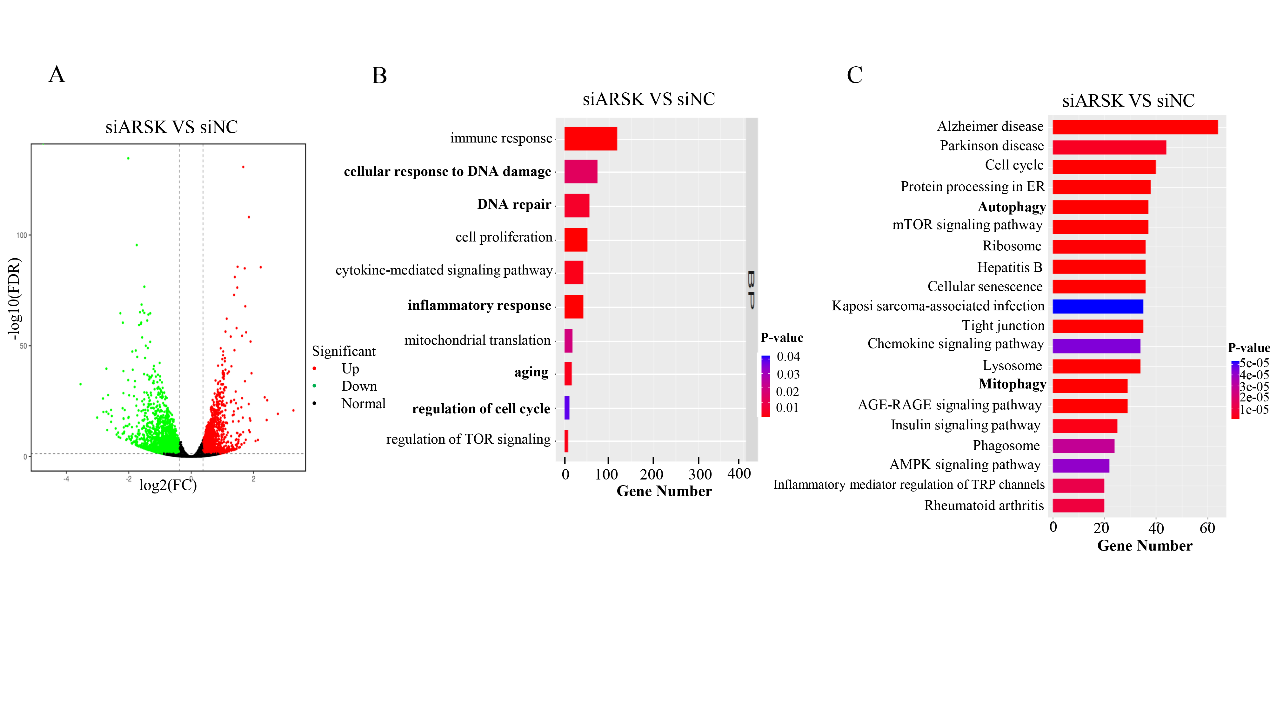
**

**Fig. S1 The result of RNA sequencing for HBE cells transfected with siRNA.**

(**A**) Volcano plot showed the DEGs in HBE cells transfected with siARSK compared with siNC (n=3 per group). (**B and C**) The results of GEO and KEGG enrichment analysis of DEGs.

**
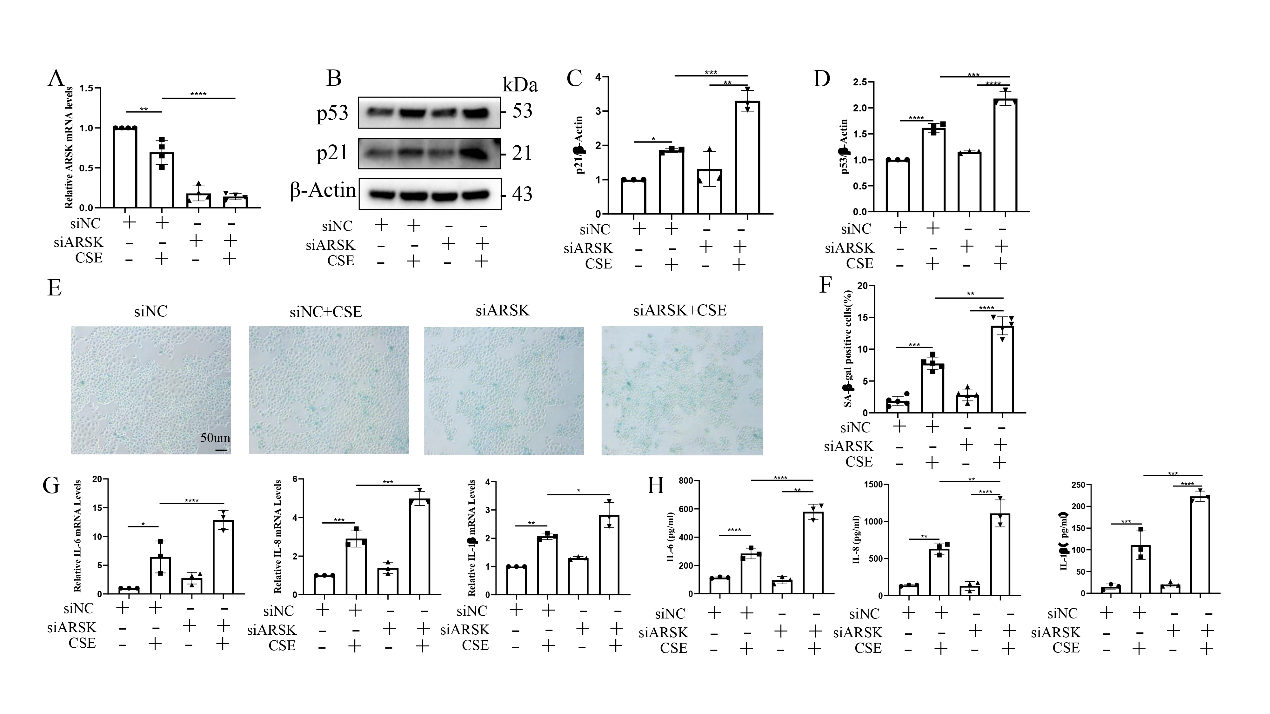
Fig. S2 The knockdown of ARSK aggravated cellular senescence induced by CSE in HBE cells.**

(**A**) The relative ARSK mRNA levels after transfection of siRNA(n=4). (**B-D**) Western blot analysis of p21 and p53 expressions in HBE cells after the knockdown of ARSK. (**E and F**) Representative images of SA-β-gal staining and percentages of SA-β-gal positive cells for HBE cells with ARSK knockdown. Scale bar=50um, magnification=200x. (**G and H**) The relative mRNA and protein levels of SASP factors in CSE (10%)-treated HBE cells with ARSK knockdown. Data were expressed as mean (SD). P values were calculated using one-way ANOVA followed by Newman-Keuls test or Students-unpaired t test. *P<0.05, **P<0.01, ***P<0.001, ****P<0.0001; SASP: senescence associated secretory phenotype.

**
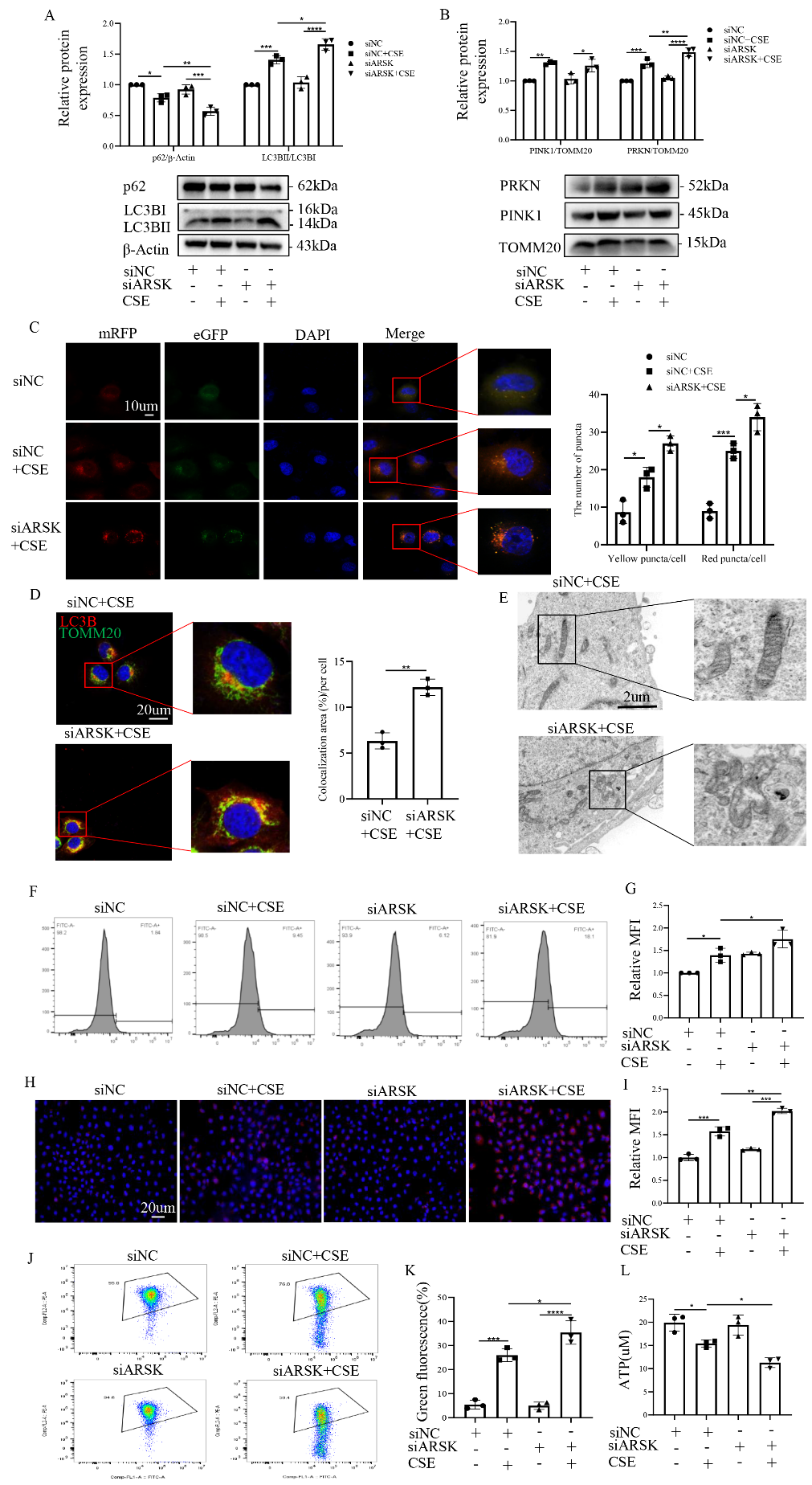
**

**Fig.S3. The knockdown of ARSK exacerbated CSE-induced exceesive mitophagy and mitochondrial dysfunction in HBE cells.**

(**A and B**) Western blot analysis of mitophagy-related proteins in CSE (10%)-treated HBE cells after siARSK transfection. (**C**) Confocal images of HBE cells with infection of mRFP-eGFP-LC3B adenovirus, along with the quantitative data of yellow and red puncta. Scale bar=10um，magnification=1000x. (**D**) Confocal images of immunostaining for LC3B and TOMM20, along with relative semiquantitative result. Red color-LC3B, Green color-TOMM20, Blue color-DAPI, Scale bar=20um, magnification=1000x. (**E**) Representative electron microscopic images of CSE-stimulated HBE cells with or without ARSK knockdown. Scale bar=2um. (**F and G**) Representative images of intracellular ROS levels and mean fluorescence intensity for CSE (10%)-induced HBE cells after knockdown of ARSK. (**H and I**) Representative images of Mitosox staining for treated and untreated HBE cells. Red color-Mitosox, Blue color-DAPI. Scale bar=20um, magnification=400x. (**J and K**) The mitochondrial membrane potential of HBE cells measured by flow cytometry and statistical graph of percentage for green fluorescence. (**L**) The levels of ATP content in HBE cells under CSE (10%) treatment after the transfection of siARSK. Data were expressed as mean (SD). P values were calculated using one-way ANOVA followed by Newman-Keuls test or Students-unpaired t test. *P<0.05, **P<0.01, ***P<0.001, ****P<0.0001.

**
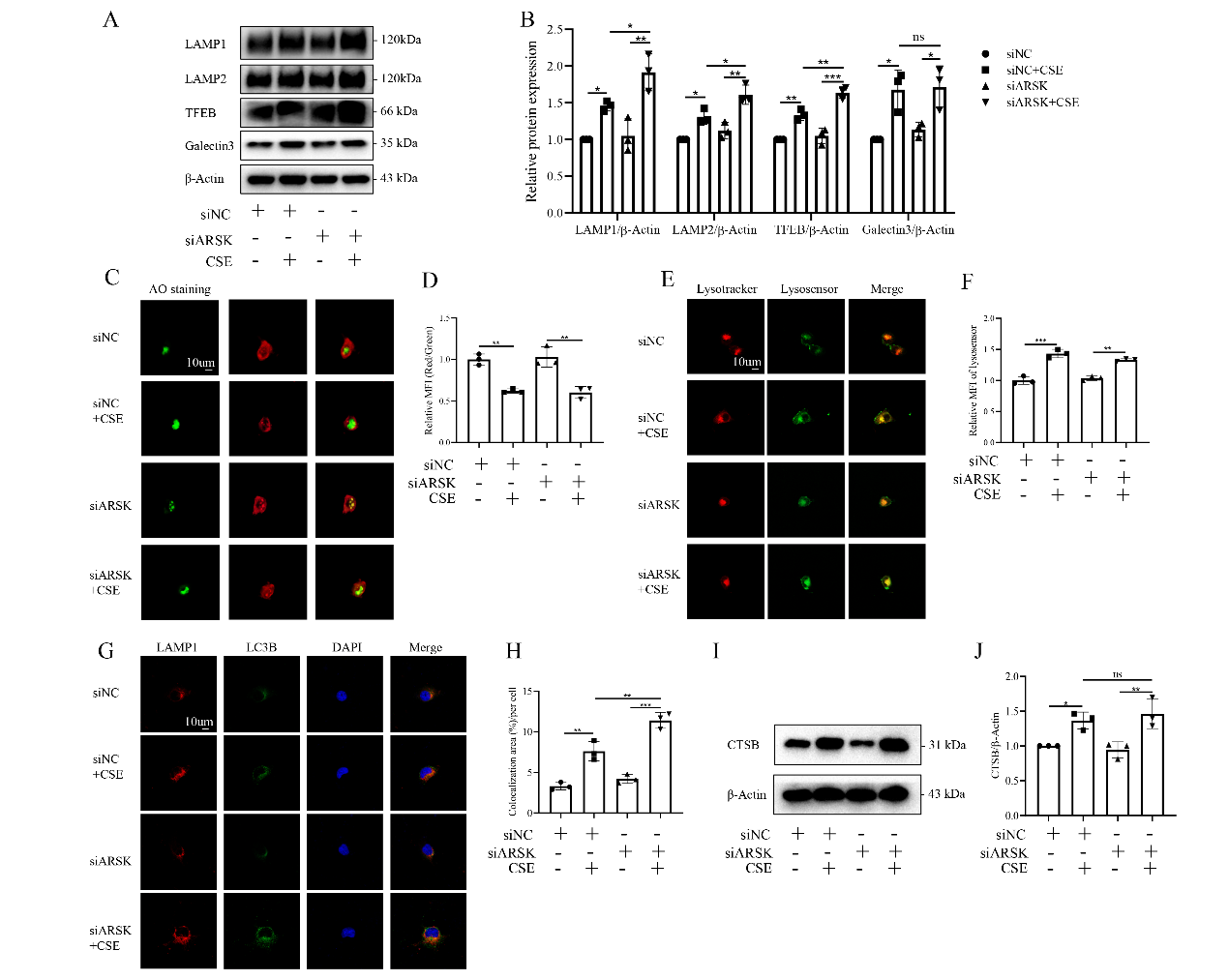
**

**Fig.S4. The lysosomal function of HBE cells after the transfection of ARSK siRNA.**

(**A and B**) Western blot analysis of lysosome biogenesis-related proteins and Galectin3 in CSE (10%)-treated HBE cells after siARSK transfection. (**C and D**) Representative images of Acridine orange staining of HBE cells, along with quantitative data of mean fluorescence intensity (MFI). Scale bar=10um, magnification=1000x. (**E and F**) Confocal images of HBE cells stained with lysotracker and lysosensor, with quantitative data of relative MFI of lysosensor. Scale bar=10um, magnification=1000x. (**G and H**) Confocal images of immunostaining for LC3B and LAMP1, along with relative semiquantitative result. Scale bar=10um, magnification=1000x.（**I and J**）Western blot analysis of CathepsinB (CTSB) in CSE (10%)-treated HBE cells after siARSK transfection. Data were expressed as mean (SD). P values were calculated using one-way ANOVA followed by Newman-Keuls test. *P<0.05, **P<0.01, ***P<0.001; ns: no significance.

**
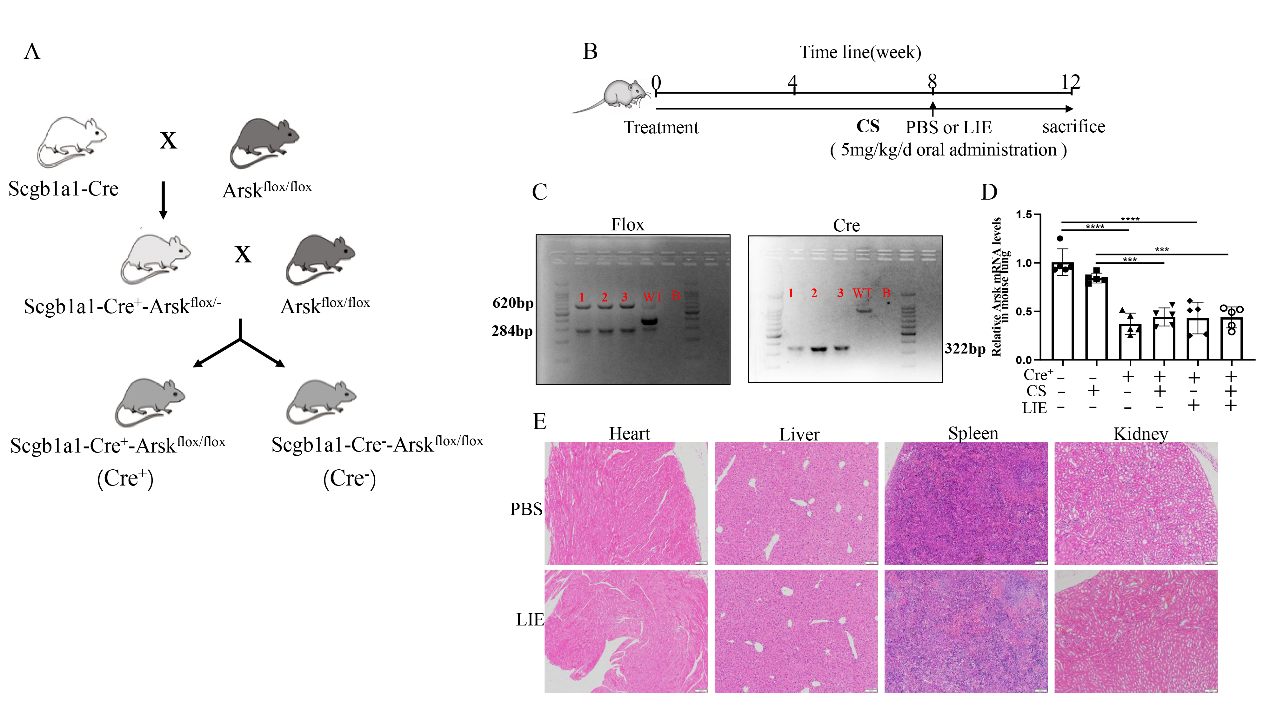
Fig. S5 The experimental schedule and identification of Arsk AEC-KO mice.**

(**A**) Generation of Arsk AEC-KO mice. (**B**) Experimental schedule. (**C**) Representative images of genotyping of Arsk AEC-KO mice. (**D**) Relative Arsk mRNA levels in mouse lung. (**E**) Representative images of HE-stained heart, liver, spleen and kidney paraffin sections from mice with oral gavage of PBS or liensinine (LIE). Data were expressed as mean (SD). P values were calculated using one-way ANOVA followed by Newman-Keuls test. *P<0.05, **P<0.01, ***P<0.001, ****P<0.0001.

**Table S1** Clinical characteristics of study subjects

|  | **Non-smoker**  (n=18) | **Healthy smoker**  (n=18) | **COPD**  (n=20) |
| --- | --- | --- | --- |
| **Age** | 58.0 (13.1) | 62.8(10.2) | 63.8(6.3) |
| **Gender (M/F)** | 18/0 | 18/0 | 20/0 |
| **BMI (kg/m^2^)** | 22.6(1.7) | 22.9(1.8) | 22.3(1.5) |
| **Smoking (pack-yrs)** | 0 | 22.2(10.6) * | 21.3(9.0) * |
| **FEV1% predicted** | 101.6(13.3) | 96.8(19.2) | 79.9(28.0) *^#^ |
| **FEV1/FVC** | 79.1(6.4) | 75.3(3.6) | 59.1(10.3) *^#^ |

Data are expressed as mean (SD). *p<0.05 vs patients in Non-smoker group. ^#^p<0.05 vs patients in healthy smoker group. COPD, chronic obstructive pulmonary disease; M/F, male/female; BMI, body mass index; FVC, forced vital capacity; FEV1, forced expiratory volume in one second.

**Table S2** Primer information for qPCR

|  | **Primer pairs (5’-3’)** |
| --- | --- |
| Human ARSK | TTAATTCATCTGGATCCGAGGAAAG |
|  | AATCGTGTGGAAGCTCG |
| Human ARSJ | CCTGTGTCTGTCCTGGAAAGA |
|  | AGATCCGTGGTAACCCACATC |
| Human β-Actin | AGAAAATCTGGCACCACACCT |
| Human AR | GATAGCACAGCCTGGATAGCA  CCAGGGACCATGTTTTGCC  CGAAGACGACAAGATGGACAA |
| Human IL-6 | CTGCTGCCTTCCCTGCC |
|  | CCTCTTTGCTGCTTTCACACAT |
| Human IL-8 | AAGAAACCACCGGAAGGAAC |
|  | ACTCCTTGGCAAAACTGCAC |
| Human IL-1β | ATGATGGCTTATTACAGTGGCAA |
|  | GTCGGAGATTCGTAGCTGGA |
| Mouse Arsk | AAAGGATGCAAGTGAACCAGG |
|  | CAGATGGGTGAATTAGTGTAGGC |
| Mouse β-Actin | AGAAAATCTGGCACCACACCT |
|  | GATAGCACAGCCTGGATAGCA |
| Mouse IL-6 | TAGTCCTTCCTACCCCAATTTCC |
|  | TTGGTCCTTAGCCACTCCTTC |
| Mouse KC | GCTTGAAGGTGTTGCCCTCAG |
|  | AAGCCTCGCGACCATTCTTG |
| Mouse IL-1β | GAAATGCCACCTTTTGACAGTG |
|  | TGGATGCTCTCATCAGGACAG |
