# Supplementary material for: Arylsulfatase K attenuates airway epithelial cell senescence in COPD by regulating parkin-mediated mitophagy
Source: Redox Biol. 2025 Jul 31;86:103793. doi: 10.1016/j.redox.2025.103793 (PMC12344987; doi:10.1016/j.redox.2025.103793)
Supplement: Multimedia component 2 [file mmc2.pdf]

Fig3E

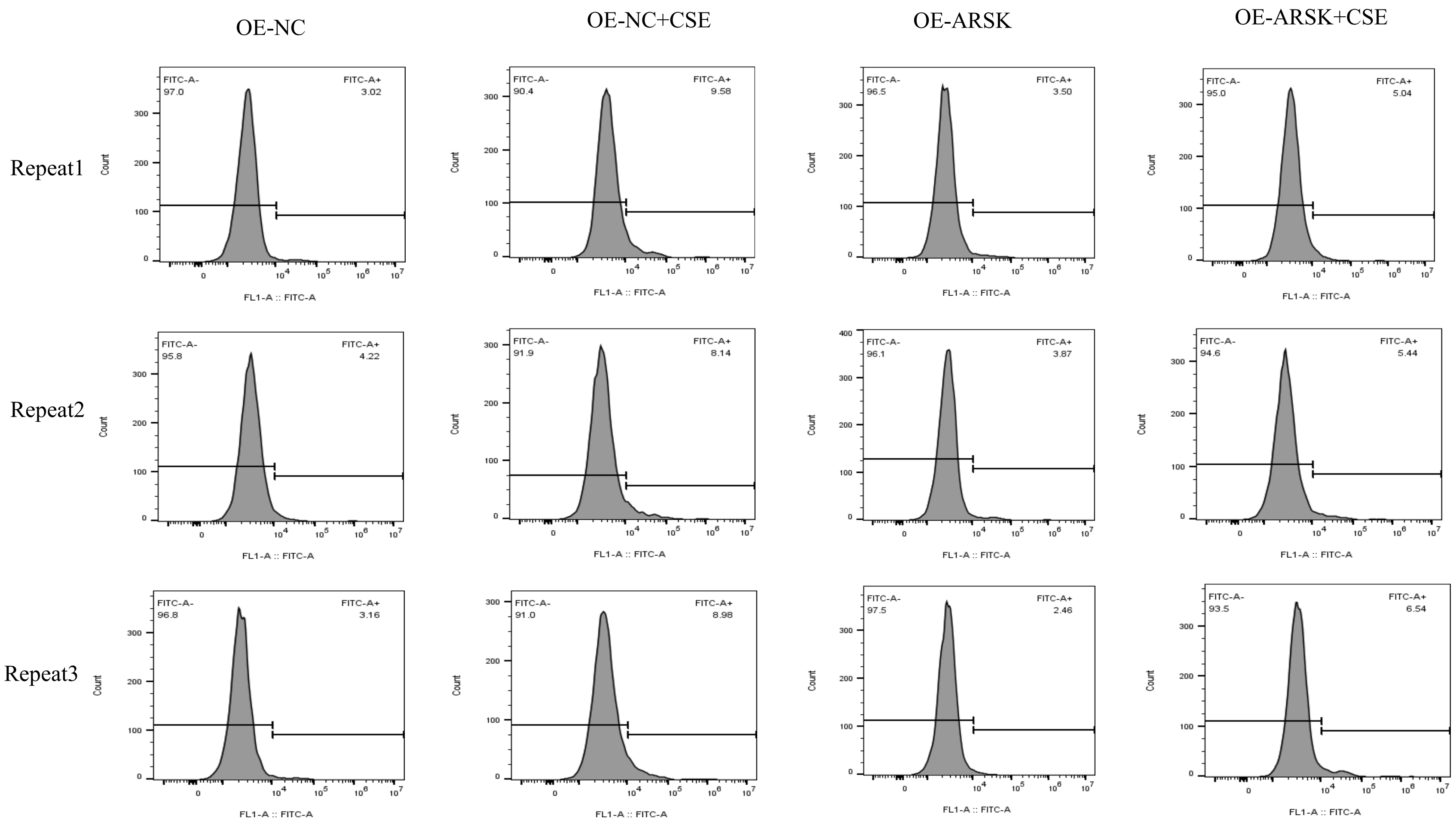
OE-NC
OE-NC+CSE
OE-ARSK
OE-ARSK+CSE
Repeat1
Repeat2
Repeat3
FITC-A- 97.0 FITC-A+ 3.02
FITC-A- 90.4 FITC-A+ 9.58
FITC-A- 96.5 FITC-A+ 3.50
FITC-A- 95.0 FITC-A+ 5.04
FITC-A- 95.8 FITC-A+ 4.22
FITC-A- 91.9 FITC-A+ 8.14
FITC-A- 96.1 FITC-A+ 3.87
FITC-A- 94.6 FITC-A+ 5.44
FITC-A- 96.8 FITC-A+ 3.16
FITC-A- 91.0 FITC-A+ 8.98
FITC-A- 97.5 FITC-A+ 2.46
FITC-A- 93.5 FITC-A+ 6.54
Count
FL1-A :: FITC-A

FigS3E

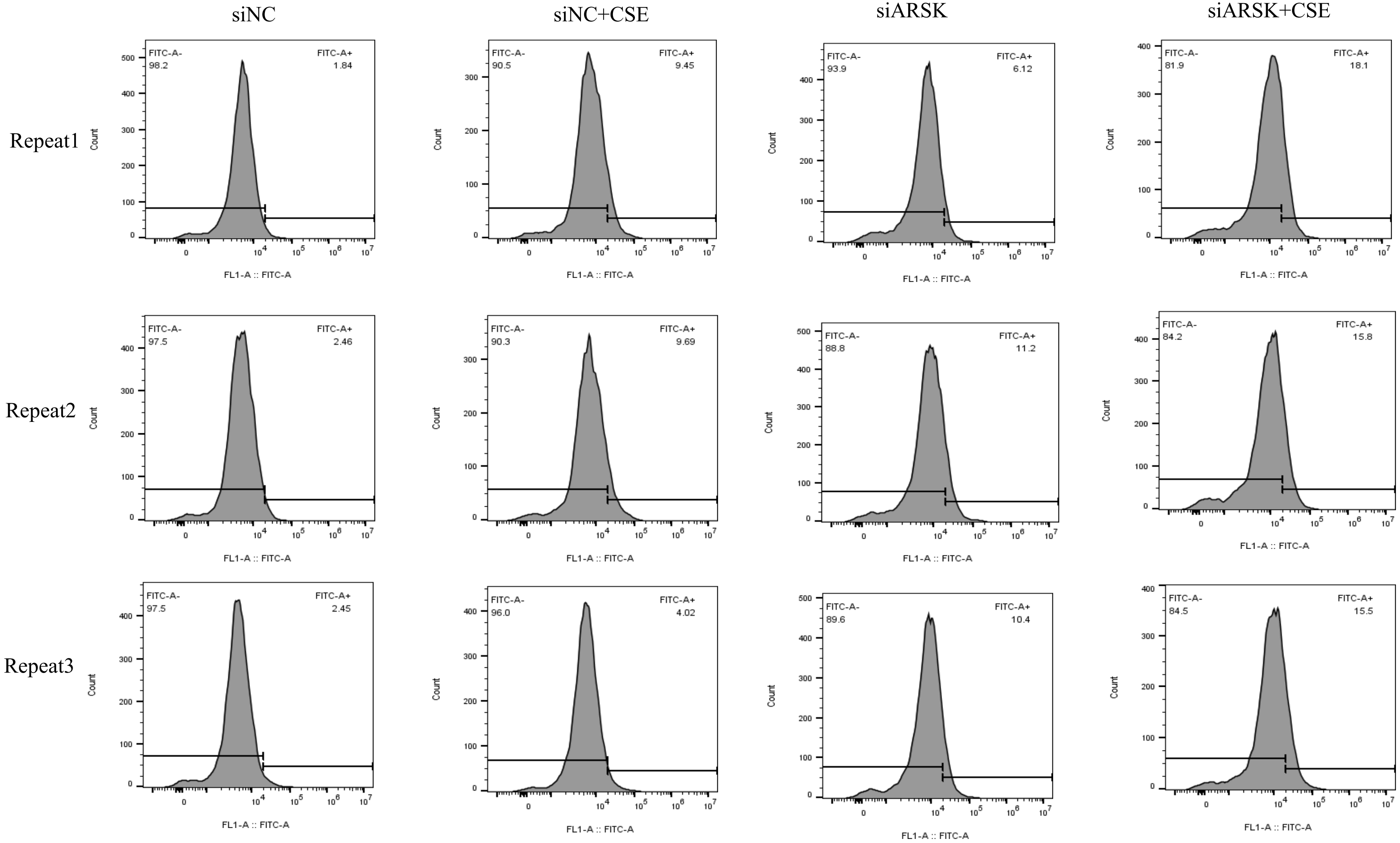
siNC
siNC+CSE
siARSK
siARSK+CSE
Repeat1
Repeat2
Repeat3
FITC-A- 98.2
FITC-A+ 1.84
FITC-A- 90.5
FITC-A+ 9.45
FITC-A- 93.9
FITC-A+ 6.12
FITC-A- 81.9
FITC-A+ 18.1
FITC-A- 97.5
FITC-A+ 2.46
FITC-A- 90.3
FITC-A+ 9.69
FITC-A- 88.8
FITC-A+ 11.2
FITC-A- 84.2
FITC-A+ 15.8
FITC-A- 97.5
FITC-A+ 2.45
FITC-A- 96.0
FITC-A+ 4.02
FITC-A- 89.6
FITC-A+ 10.4
FITC-A- 84.5
FITC-A+ 15.5
Count
FL1-A :: FITC-A

Fig4E

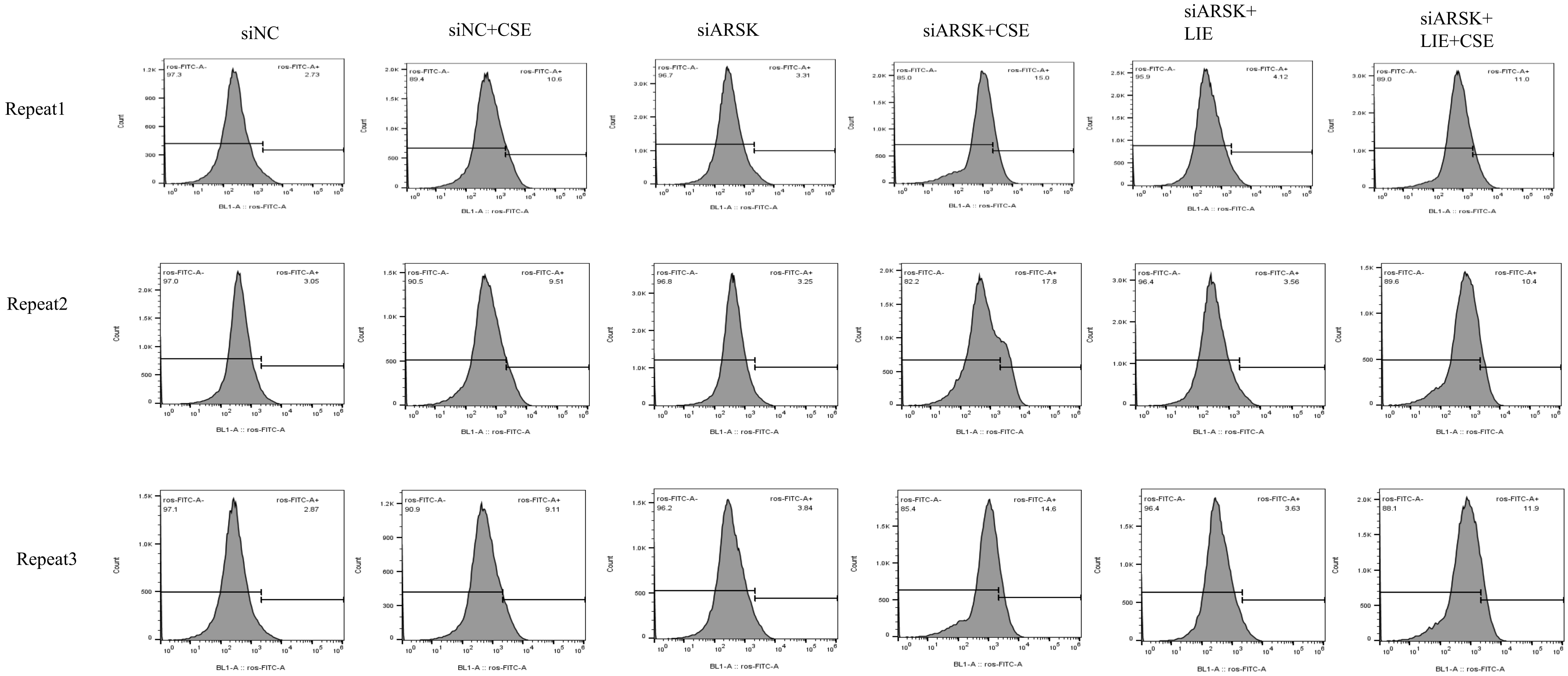
siNC
siNC+CSE
siARSK
siARSK+CSE
siARSK+ LIE
siARSK+ LIE+CSE
Repeat1
Repeat2
Repeat3
ros-FITC-A-
ros-FITC-A+
Count
BL1-A :: ros-FITC-A
97.3 2.73
89.4 10.6
96.7 3.31
85.0 15.0
95.9 4.12
89.0 11.0
97.0 3.05
90.5 9.51
96.8 3.25
82.2 17.8
96.4 3.56
89.6 10.4
97.1 2.87
90.9 9.11
96.2 3.84
85.4 14.6
96.4 3.63
88.1 11.9

Fig5H

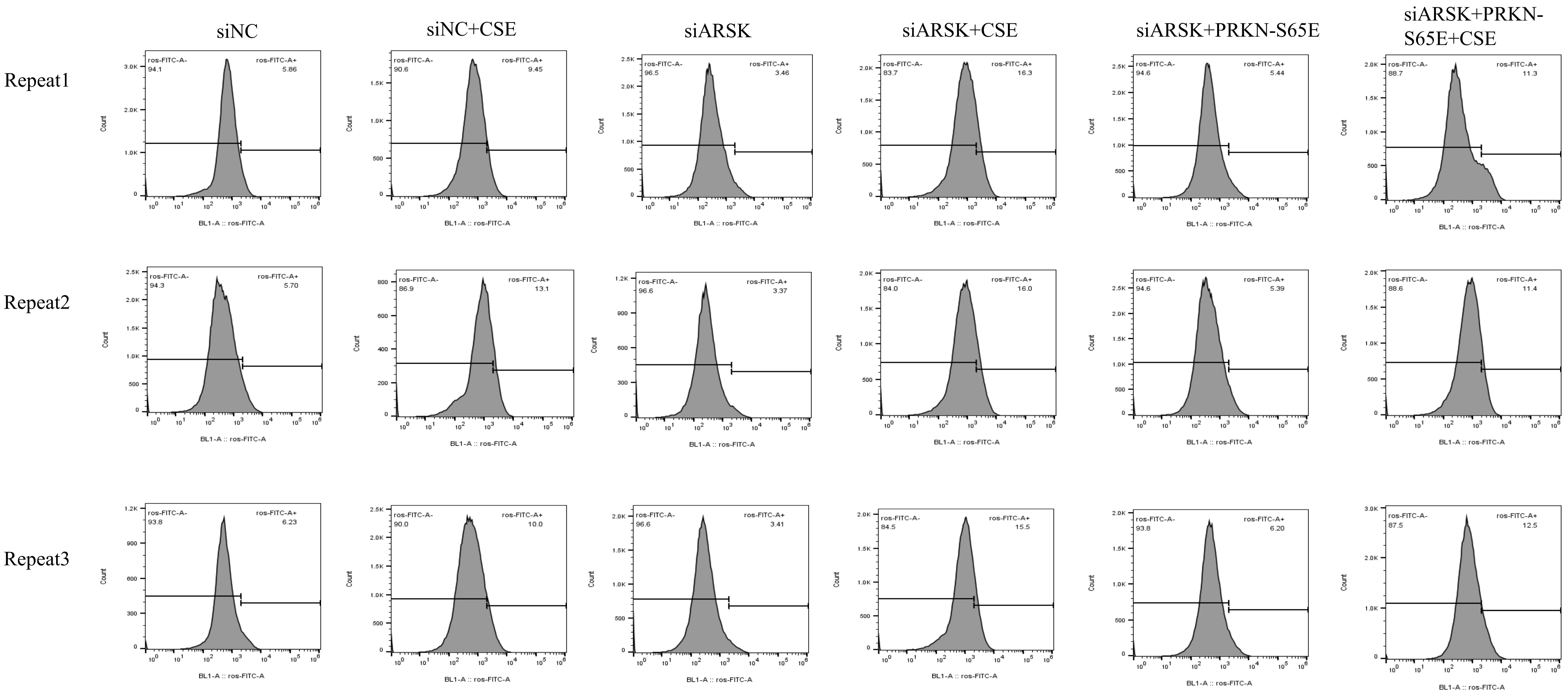
siNC
siNC+CSE
siARSK
siARSK+CSE
siARSK+PRKN-S65E
siARSK+PRKN-S65E+CSE
Repeat1
Repeat2
Repeat3
ros-FITC-A-
ros-FITC-A+
Count
BL1-A :: ros-FITC-A
94.1
5.86
90.6
9.45
96.5
3.46
83.7
16.3
94.6
5.44
88.7
11.3
94.3
5.70
86.9
13.1
96.6
3.37
84.0
16.0
94.6
5.39
88.6
11.4
93.8
6.23
90.0
10.0
96.6
3.41
84.5
15.5
93.8
6.20
87.5
12.5

Fig3H

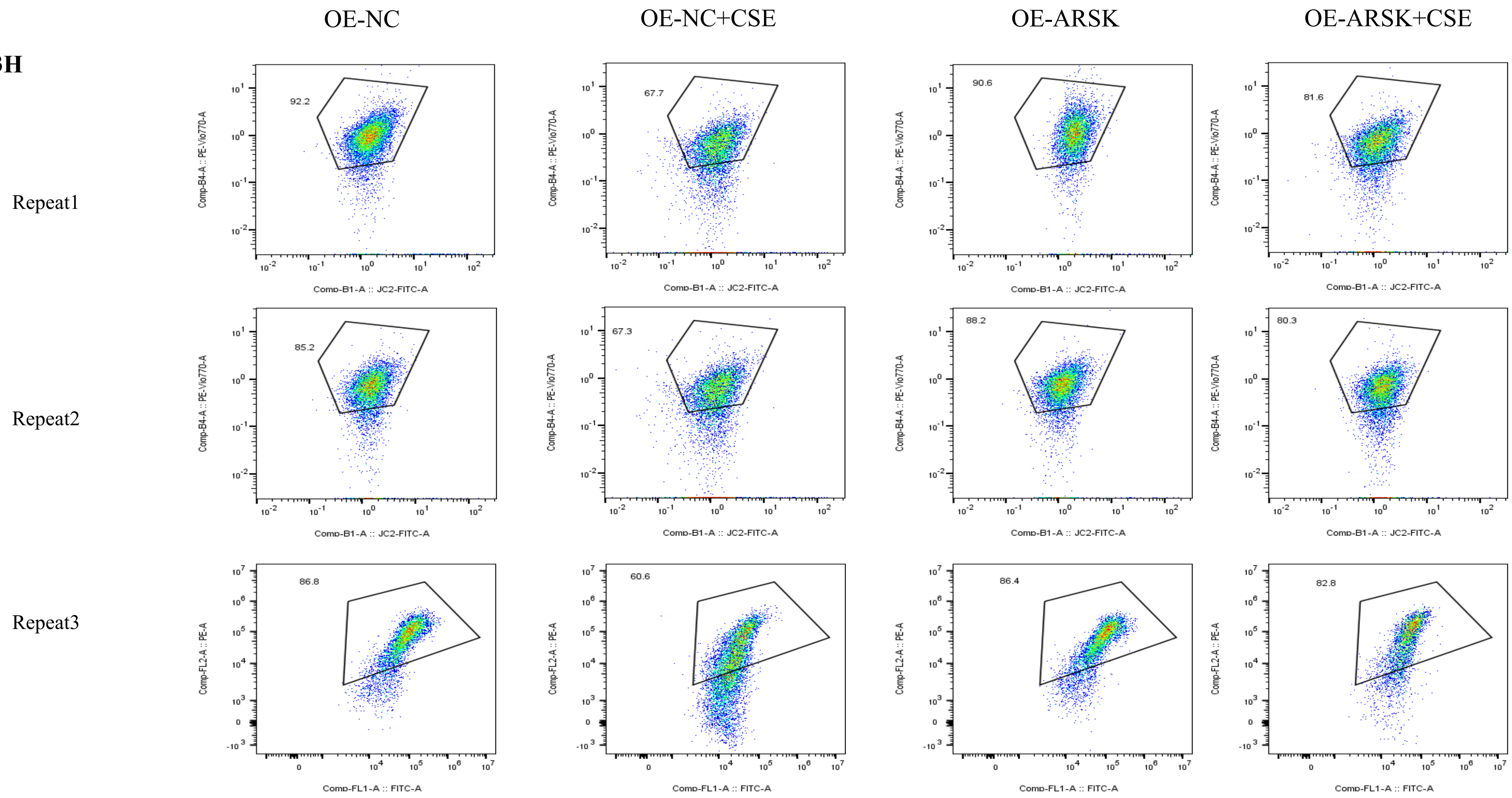
OE-NC
OE-NC+CSE
OE-ARSK
OE-ARSK+CSE
Repeat1
Repeat2
Repeat3
92.2
67.7
90.6
81.6
85.2
67.3
88.2
80.3
86.8
60.6
86.4
82.8
Comp-B4-A :: PE-Vio770-A
Comp-B1-A :: JC2-FITC-A
Comp-FL2-A :: PE-A
Comp-FL1-A :: FITC-A

FigS3H

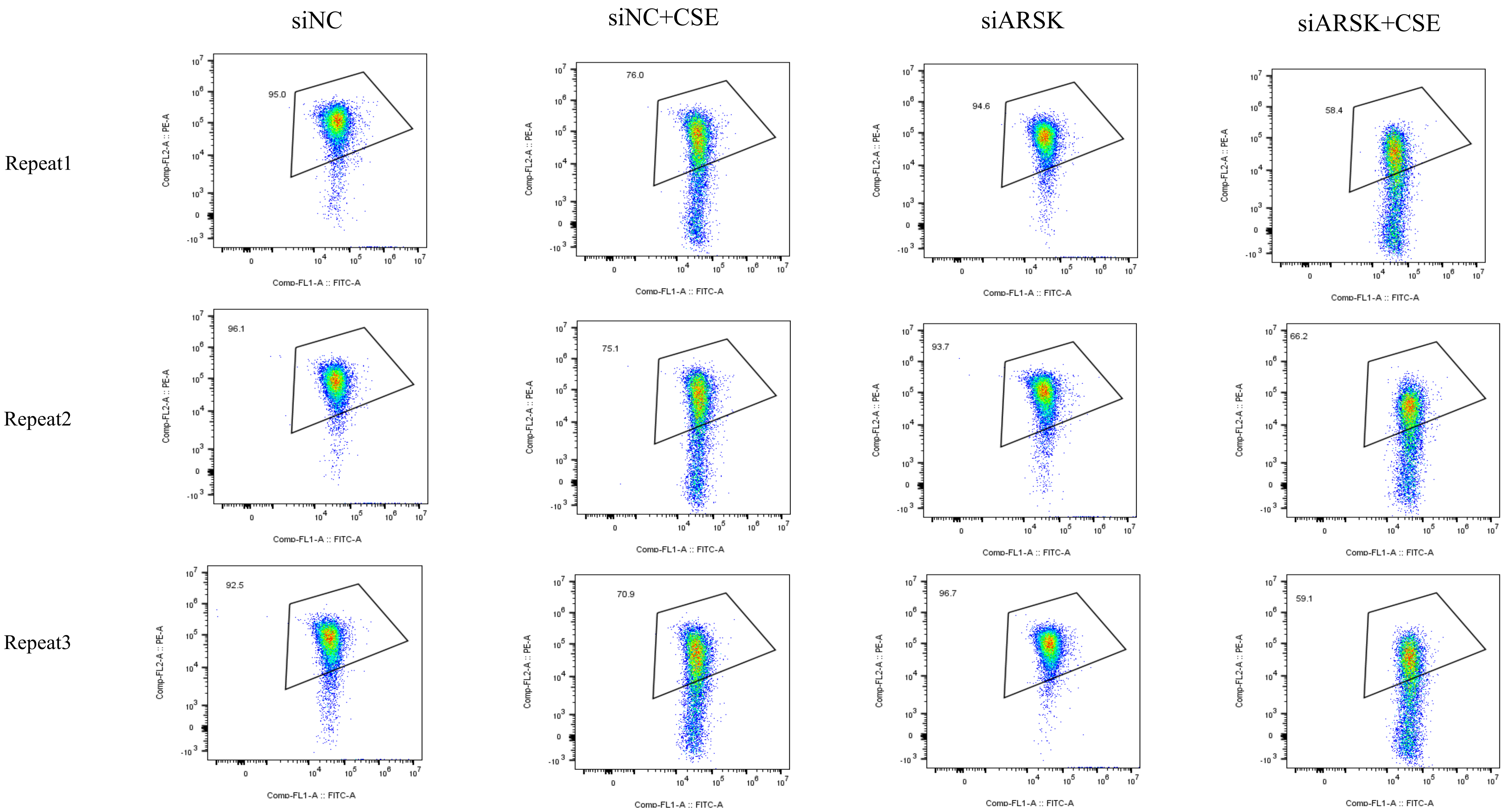
siNC
siNC+CSE
siARSK
siARSK+CSE
Repeat1
Repeat2
Repeat3
95.0
76.0
94.6
58.4
96.1
75.1
93.7
66.2
92.5
70.9
96.7
59.1
Comp-FL2-A :: PE-A
Comp-FL1-A :: FITC-A

Fig4H

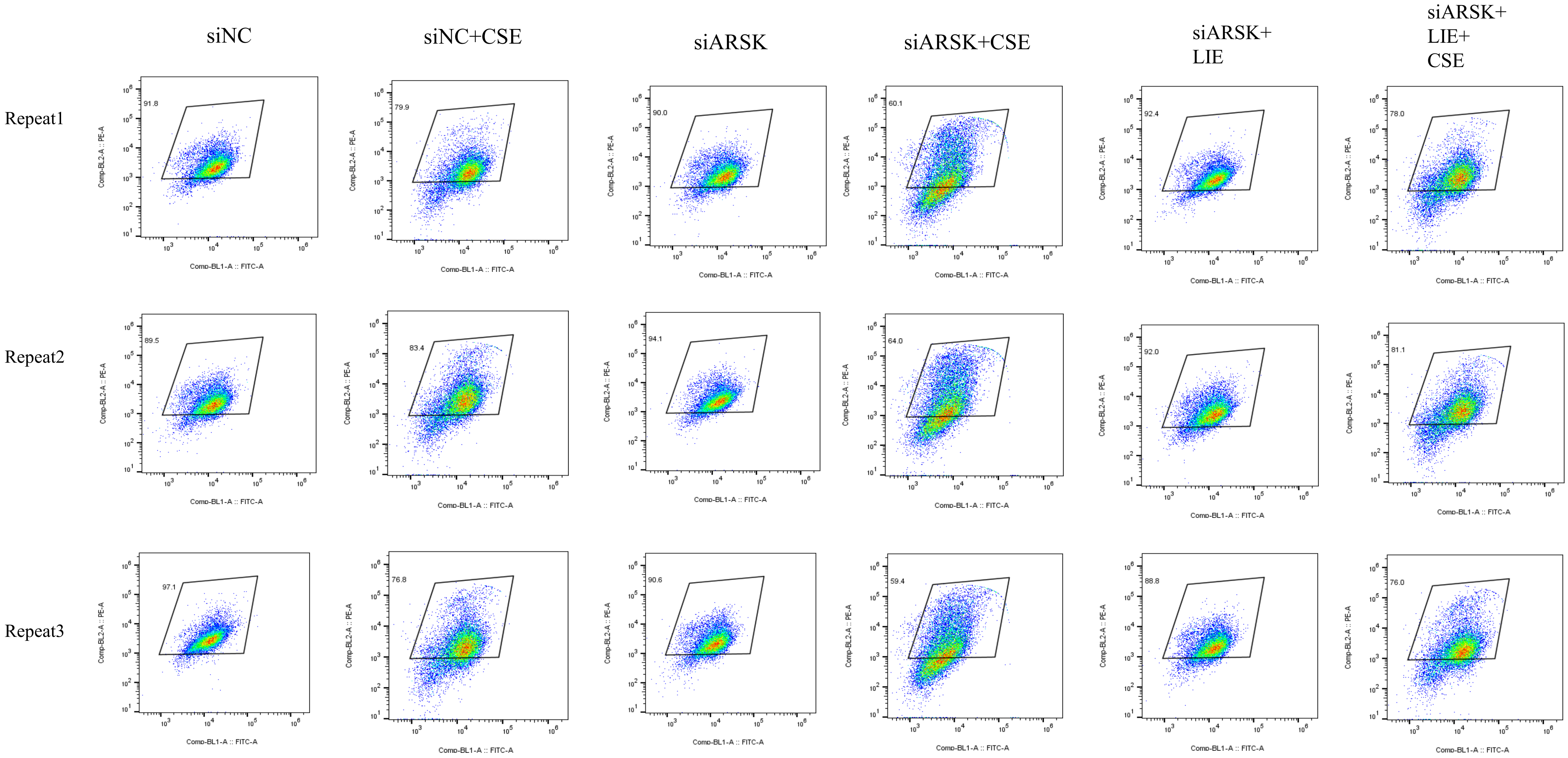
siNC
siNC+CSE
siARSK
siARSK+CSE
siARSK+ LIE
siARSK+ LIE+ CSE
Repeat1
91.8
79.9
90.0
60.1
92.4
78.0
Repeat2
89.5
83.4
94.1
64.0
92.0
81.1
Repeat3
97.1
76.8
90.6
59.4
88.8
76.0
Comp-BL2-A :: PE-A
Comp-BL1-A :: FITC-A

Fig5K

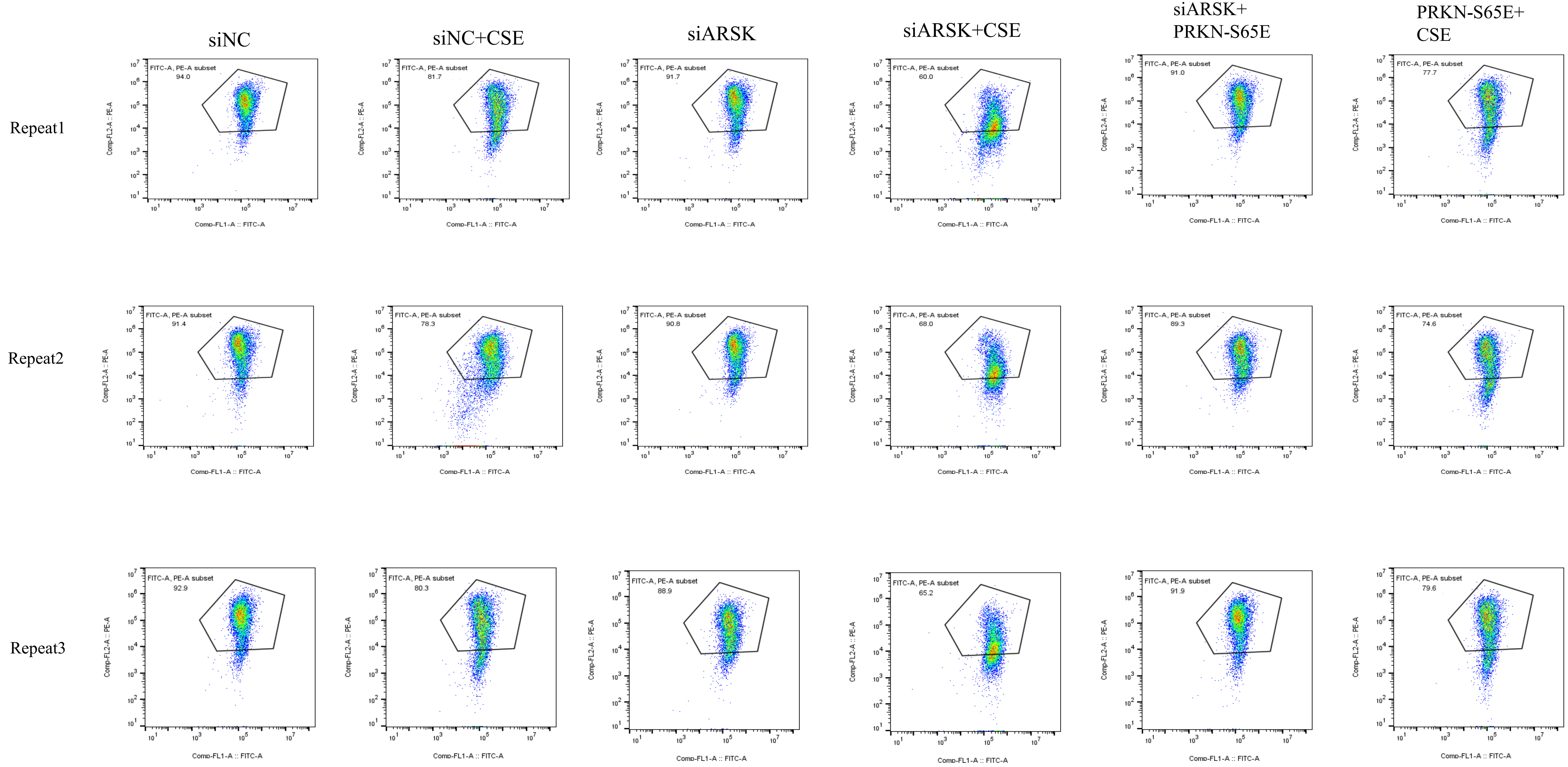

siNC
siNC+CSE
siARSK
siARSK+CSE
siARSK+ PRKN-S65E
siARSK+ PRKN-S65E+ CSE
Repeat1
Repeat2
Repeat3
FITC-A, PE-A subset
94.0
81.7
91.7
60.0
91.0
77.7
91.4
78.3
90.8
68.0
89.3
74.6
92.9
80.3
88.9
65.2
91.9
79.6
Comp-FL2-A :: PE-A
Comp-FL1-A :: FITC-A
