# Supplementary material for: Arylsulfatase K attenuates airway epithelial cell senescence in COPD by regulating parkin-mediated mitophagy
Source: Redox Biol. 2025 Jul 31;86:103793. doi: 10.1016/j.redox.2025.103793 (PMC12344987; doi:10.1016/j.redox.2025.103793)

Fig2B

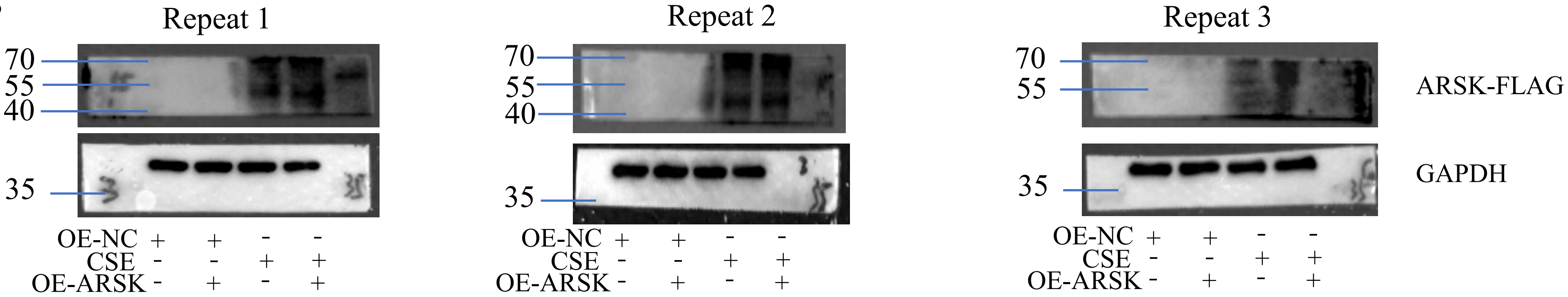

Fig2C

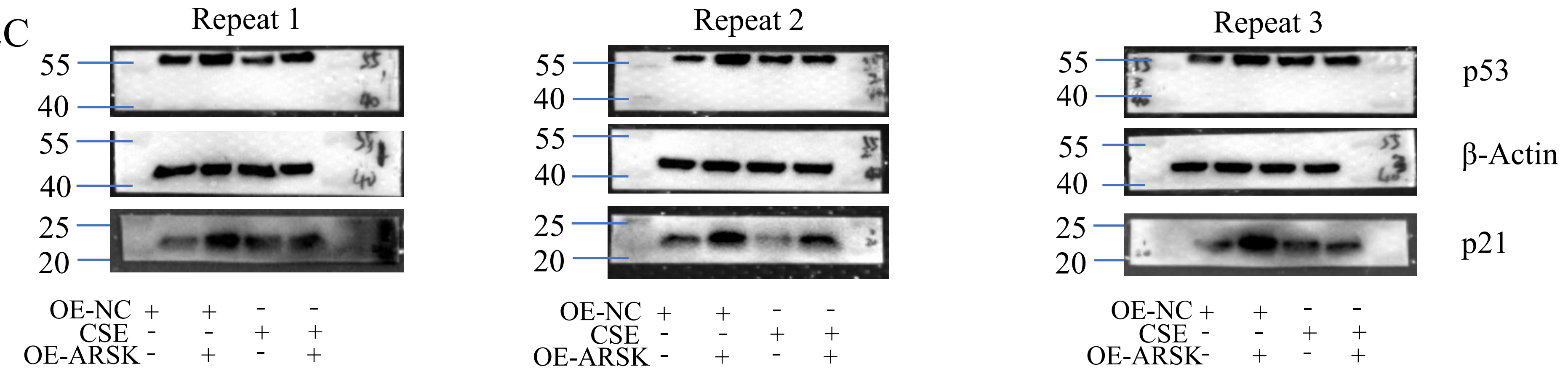

FigS2B

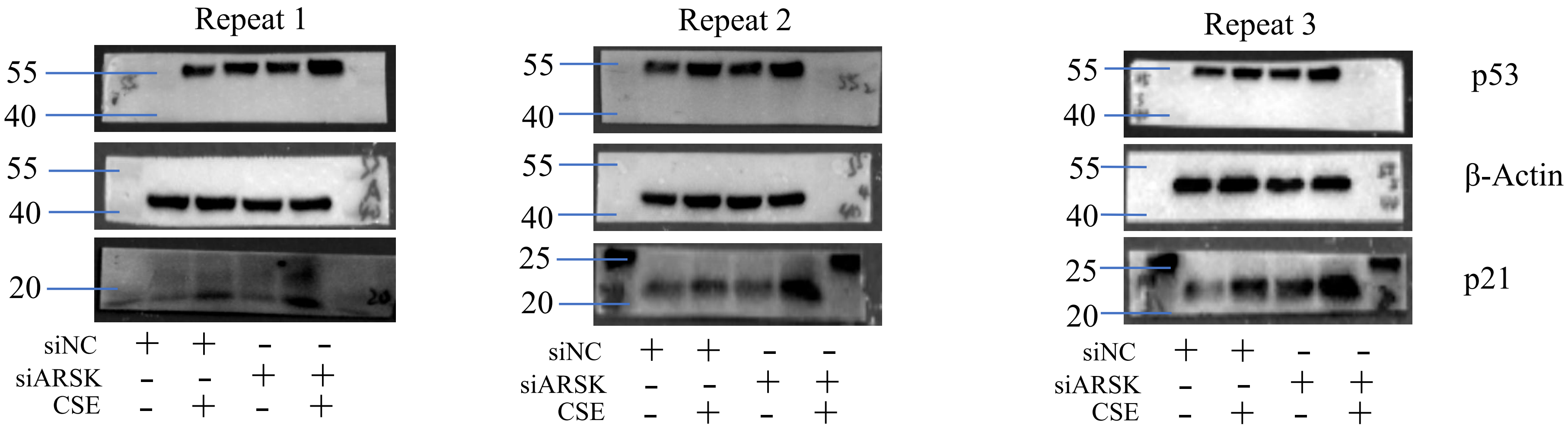

Fig3A

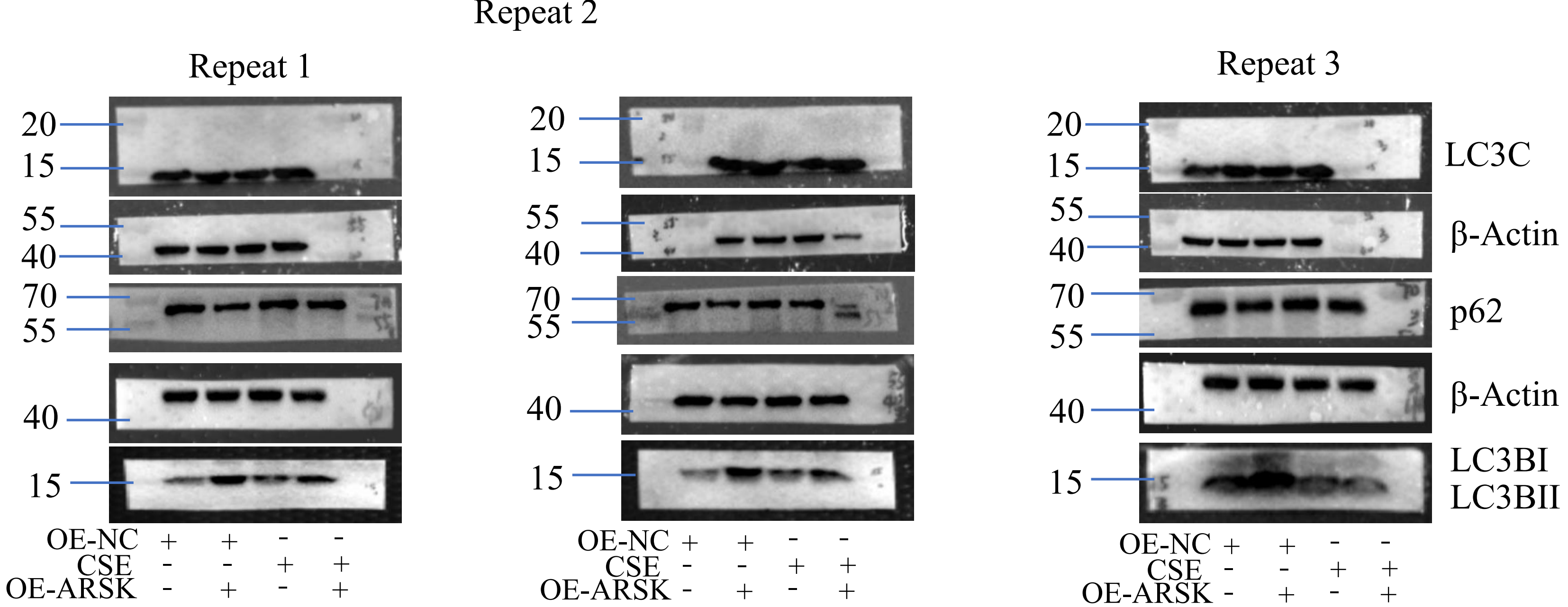
Repeat 1
Repeat 2
Repeat 3
20
15
55
40
70
55
40
15
LC3C
β-Actin
p62
β-Actin
LC3BI
LC3BII
OE-NC + + - -
CSE - - + +
OE-ARSK - + - +

Fig3B

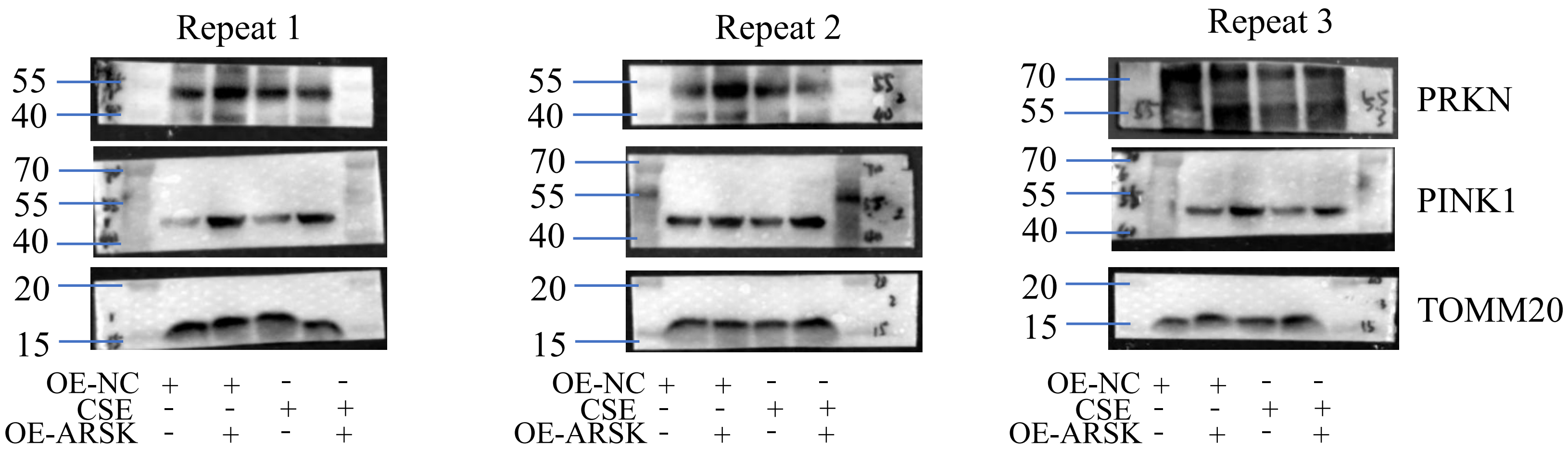
Repeat 1
Repeat 2
Repeat 3
55
40
70
55
40
20
15
PRKN
PINK1
TOMM20
OE-NC + + - -
CSE - - + +
OE-ARSK - + - +

FigS3A

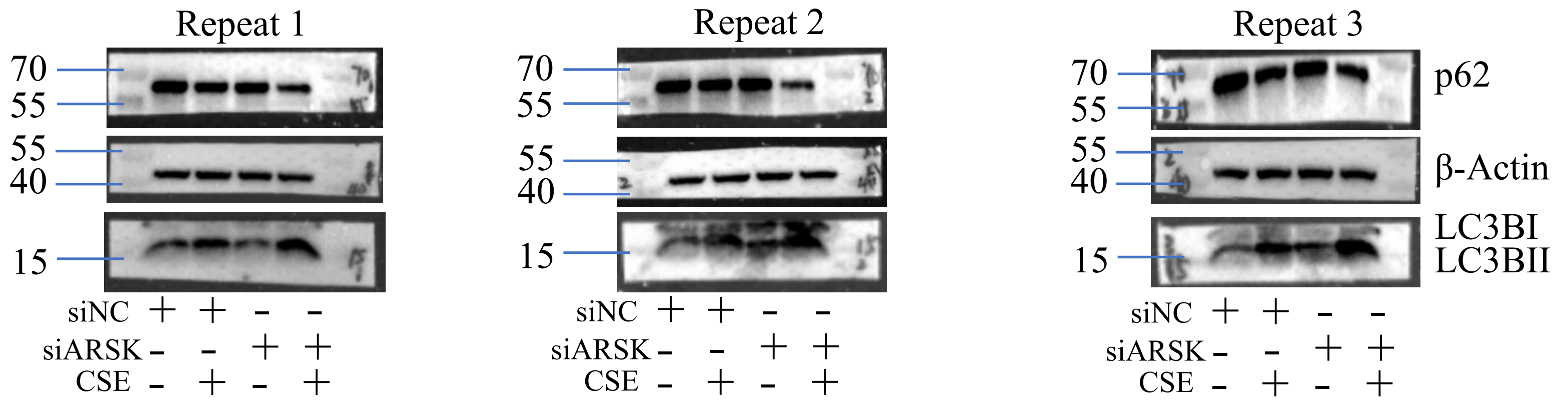

FigS3B

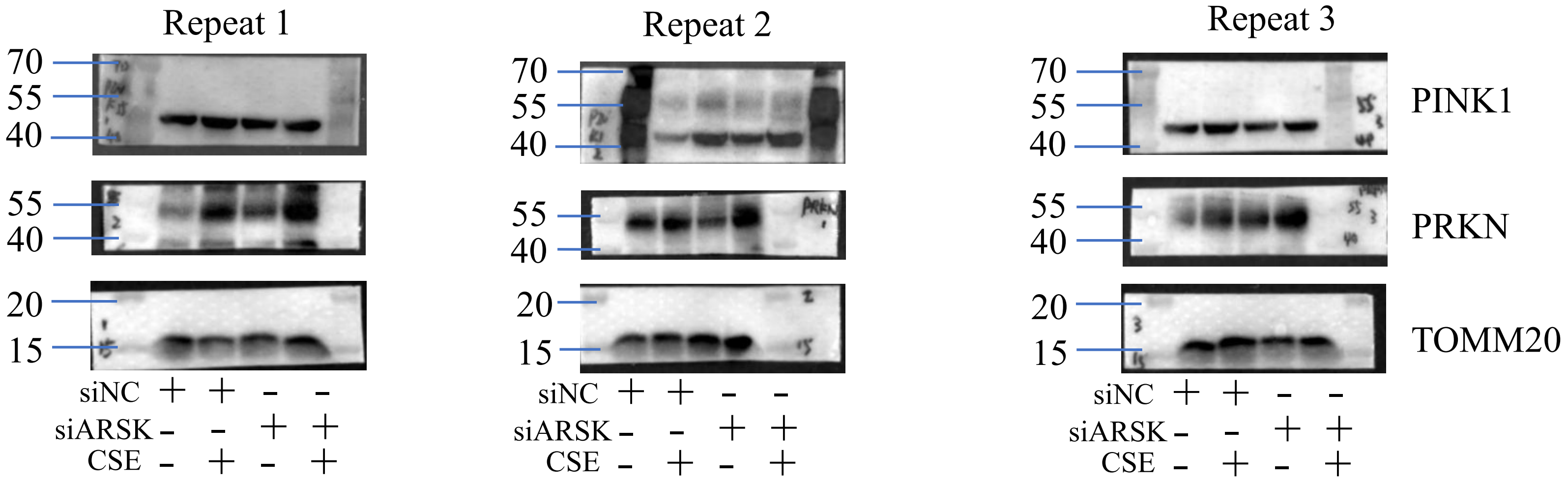

Fig4B

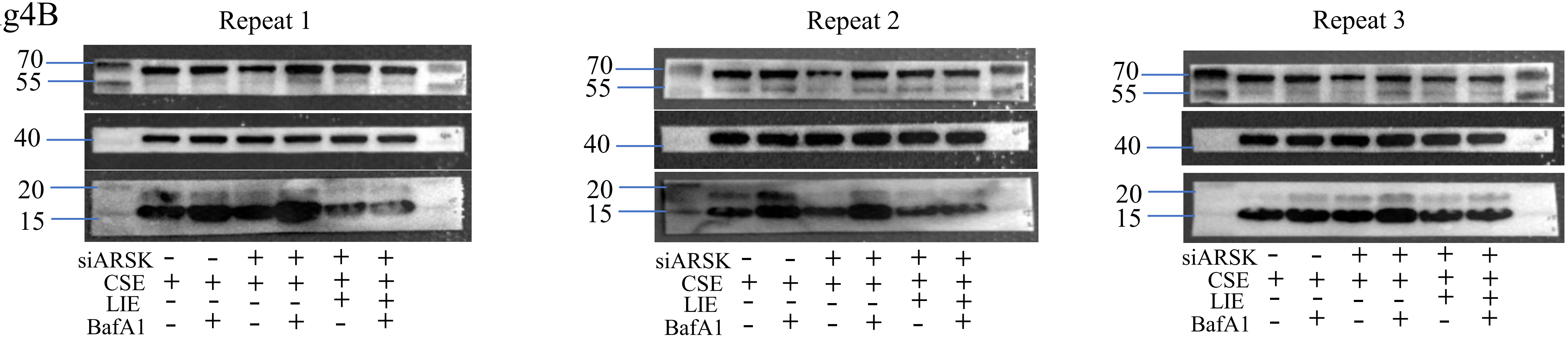

Fig4E

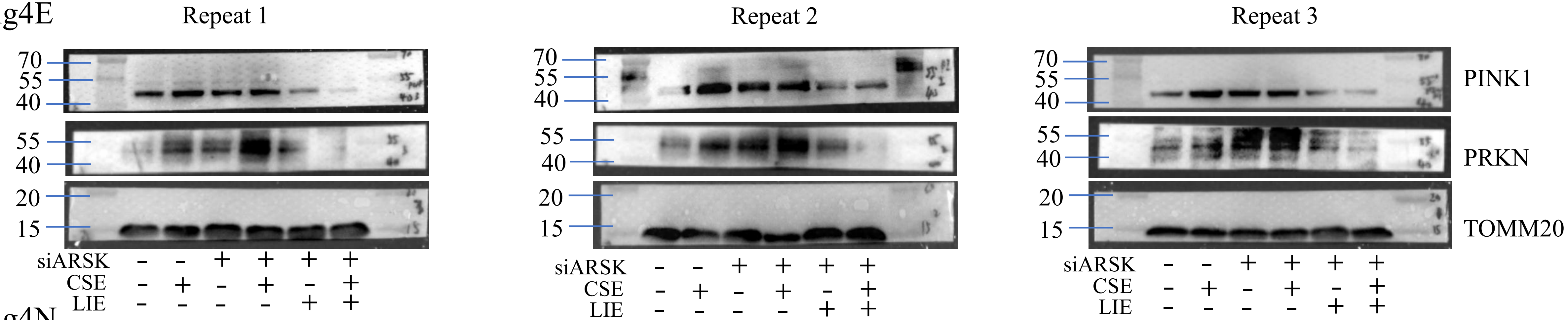

Fig4N

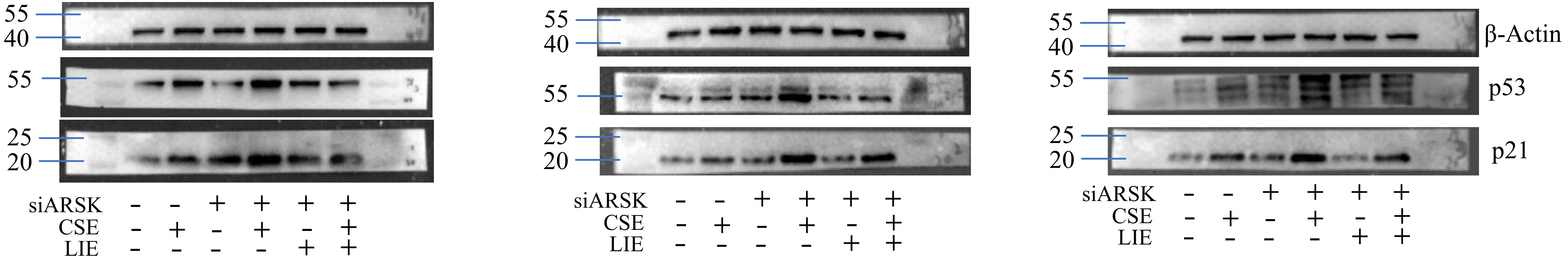

Fig5B

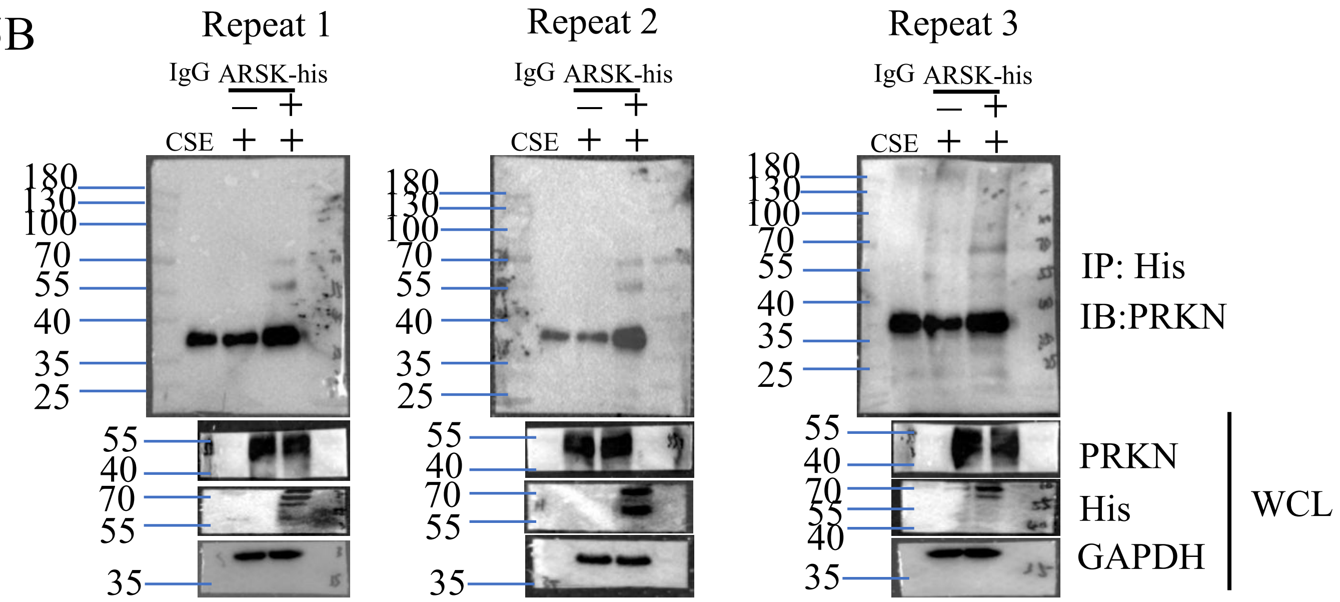

Fig5C

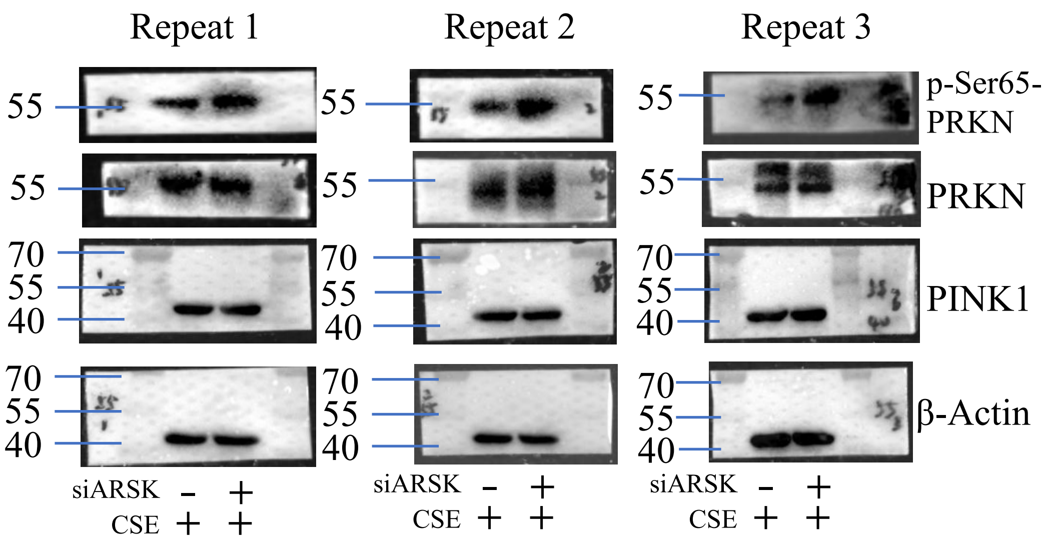

Fig5D

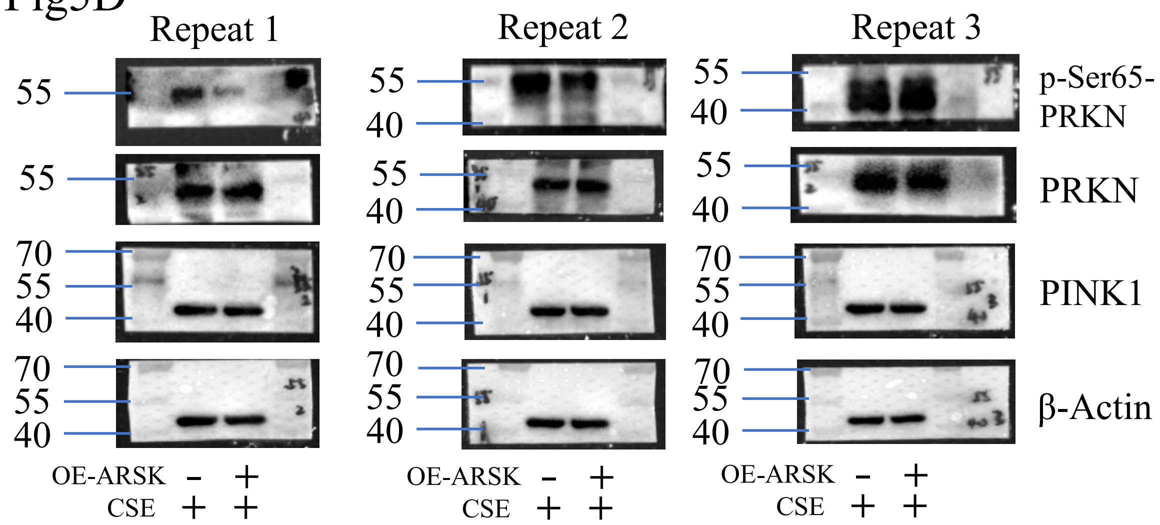

Fig5E

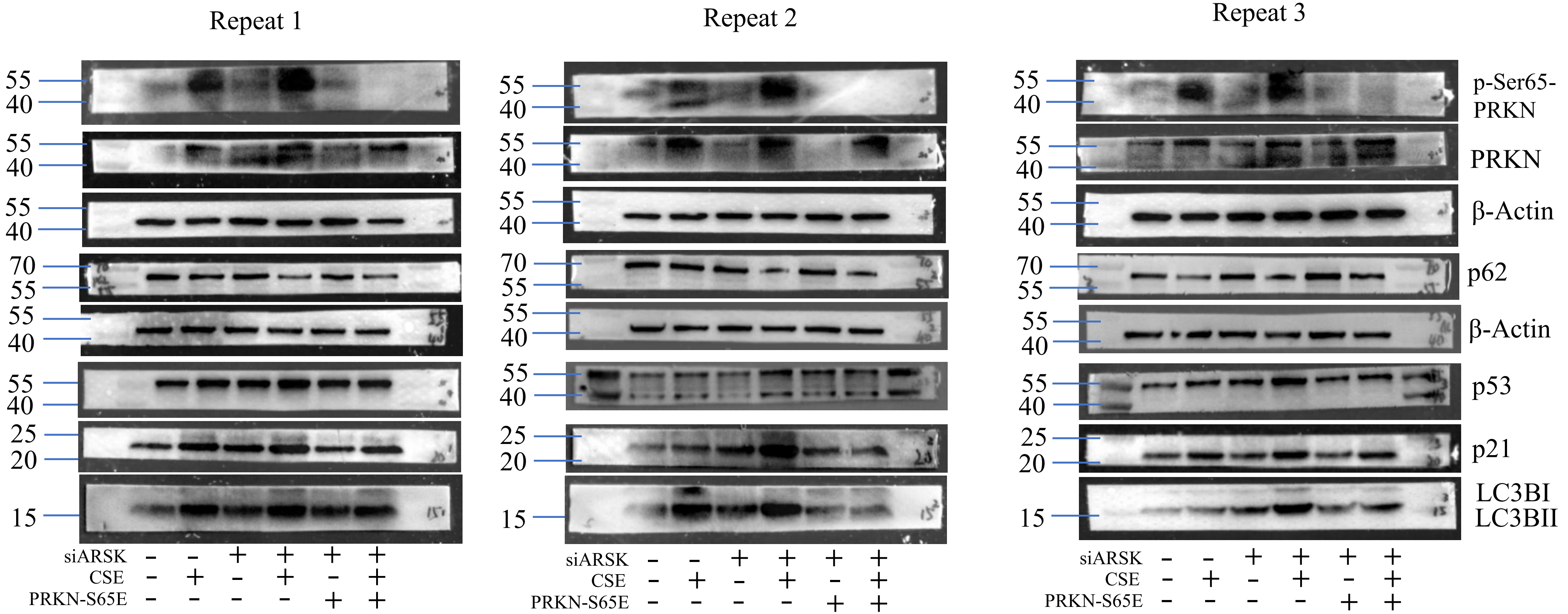

Repeat 1
Repeat 2
Repeat 3
p-Ser65-PRKN
PRKN
β-Actin
p62
β-Actin
p53
p21
LC3BI
LC3BII
siARSK - - + + + +
CSE - + - + - +
PRKN-S65E - - - - + +

Fig5G

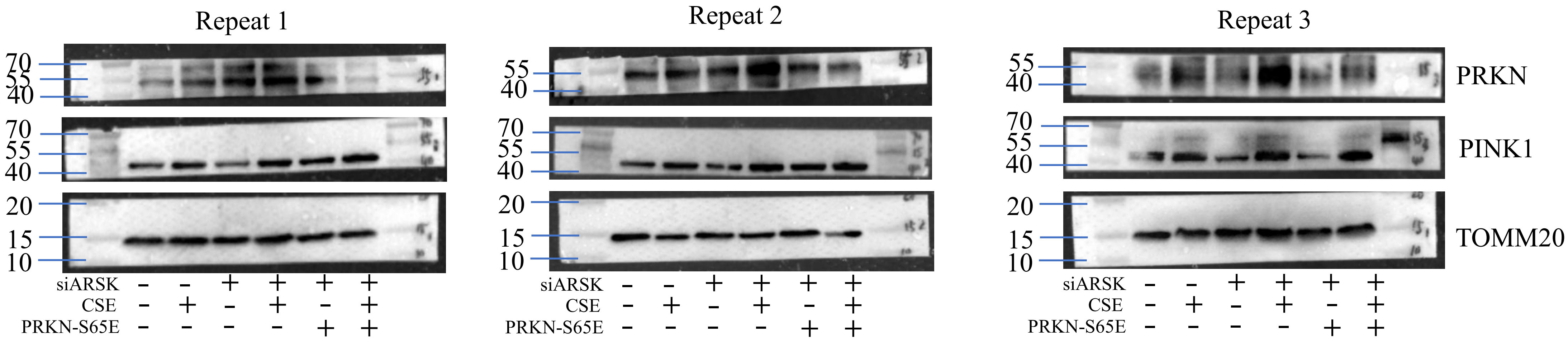

Repeat 1
Repeat 2
Repeat 3
PRKN
PINK1
TOMM20
siARSK - - + + + +
CSE - + - + - +
PRKN-S65E - - - - + +

Fig6C

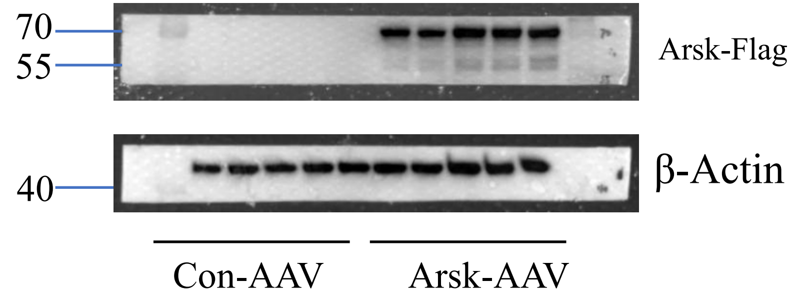
70
55
Arsk-Flag
40
β-Actin
Con-AAV
Arsk-AAV

Fig6O

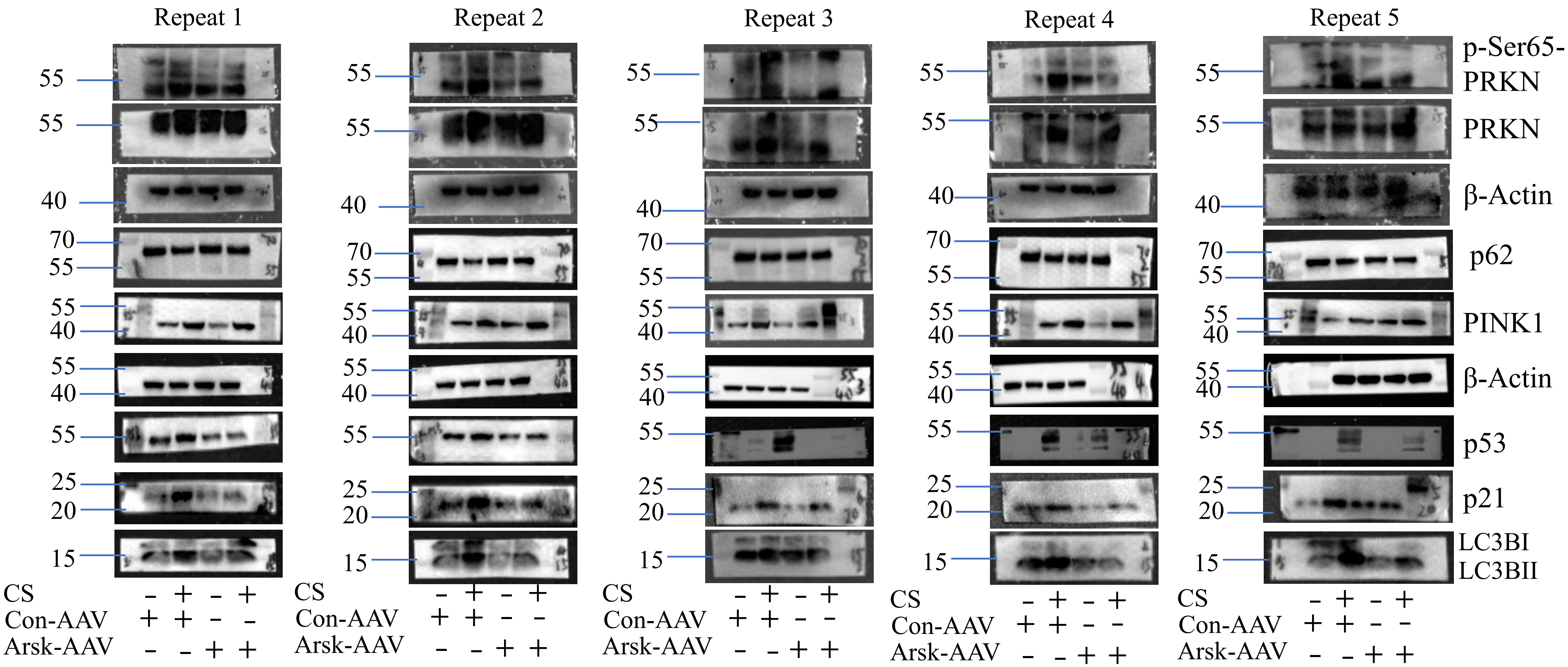
Repeat 1
Repeat 2
Repeat 3
Repeat 4
Repeat 5
p-Ser65-PRKN
PRKN
β-Actin
p62
PINK1
β-Actin
p53
p21
LC3BI
LC3BII
55
40
70
25
20
15
CS – + – +
Con-AAV + + – –
Arsk-AAV – – + +

Fig7L

Repeat 1

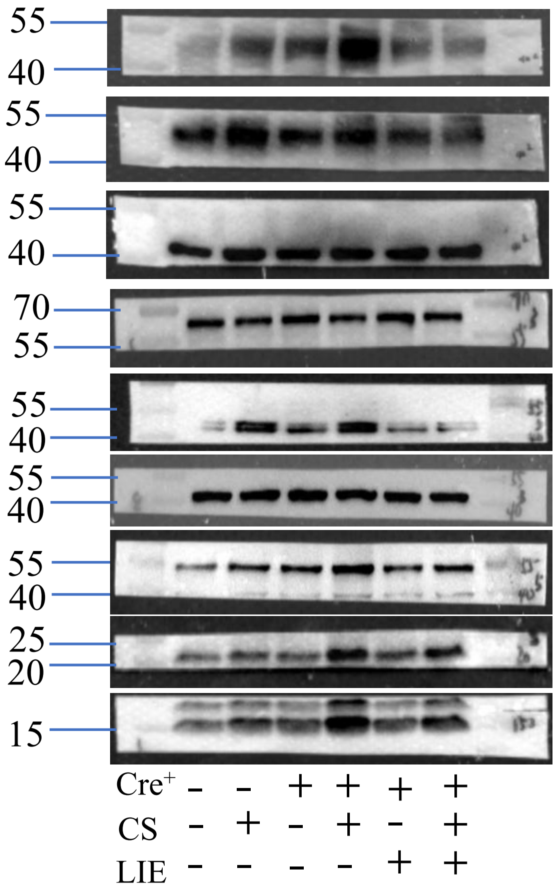

Repeat 2

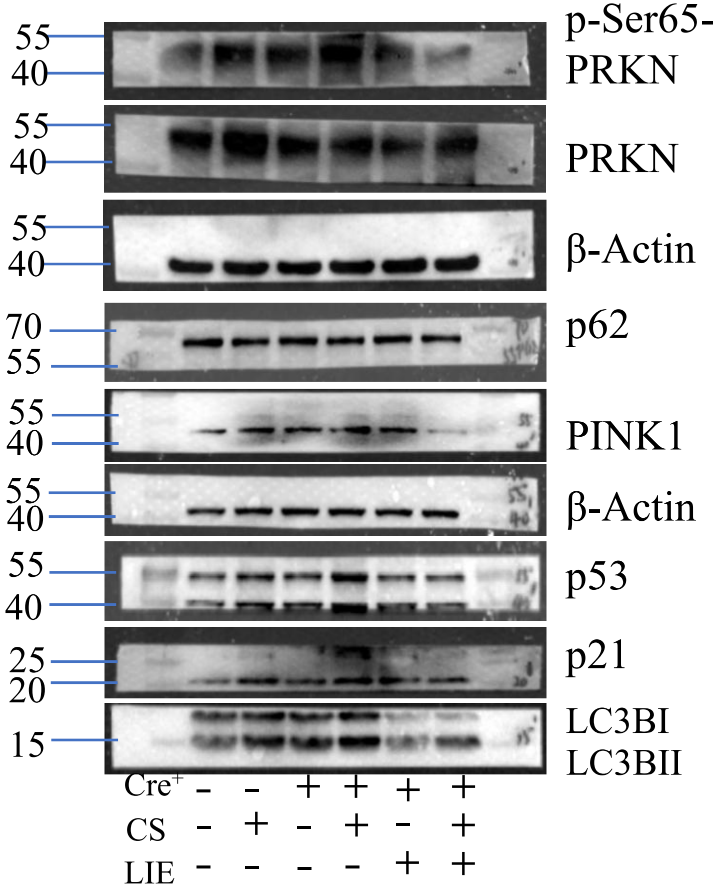

Fig7L

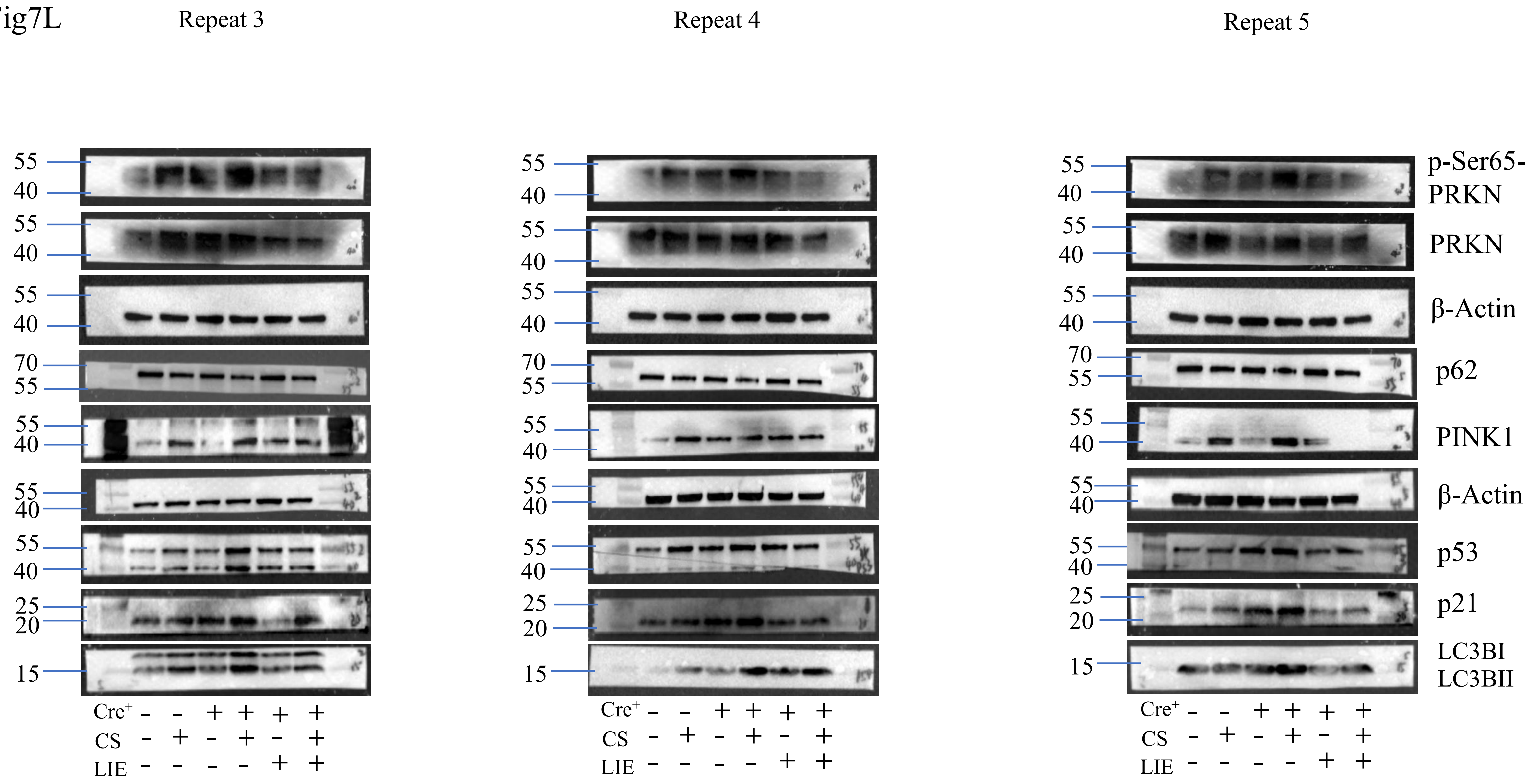
Repeat 3
Repeat 4
Repeat 5
55
40
70
25
20
15
p-Ser65-PRKN
PRKN
β-Actin
p62
PINK1
β-Actin
p53
p21
LC3BI
LC3BII
Cre+ - - + + + +
CS - + - + - +
LIE - - - - + +

FigS4A

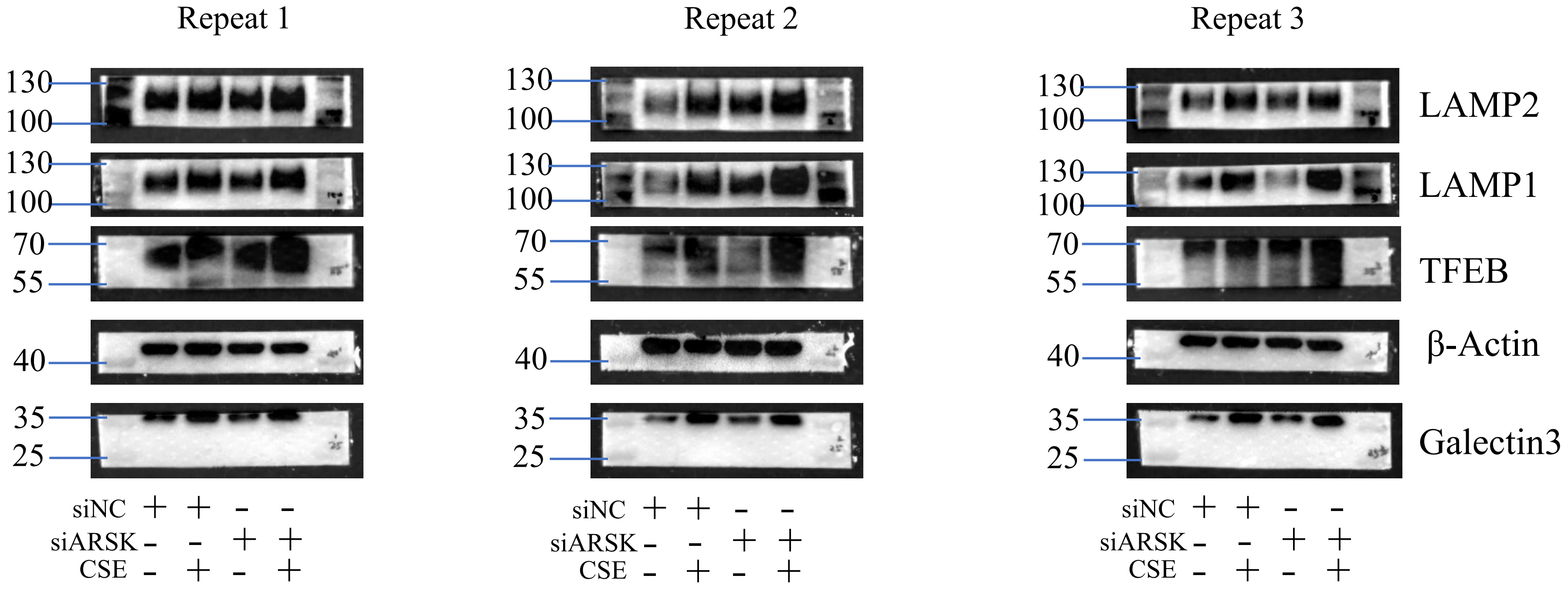

FigS4I

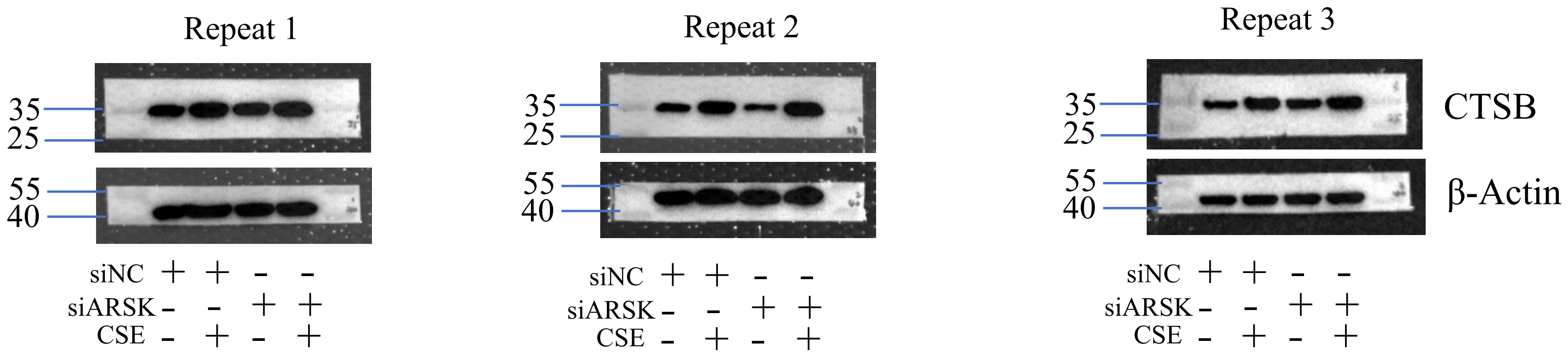

Supplement: Multimedia component 3 [file mmc3.pdf]
